# Maintenance of Auditory Deviance Processing Following Auricular Vagal Neuromodulation Therapy in Disorders of Consciousness

**DOI:** 10.3390/brainsci16101072

**Published:** 2026-10-06

**Authors:** Samuel López-Rodríguez, Barbara Postigo-Alonso, Myrtha O’Valle-Rodríguez, Marta Gómez-Herranz, María Dolores Navarro-Pérez, Joan Ferri-Campos, Enrique Noé-Sebastián, Alejandro Galvao-Carmona

**Affiliations:** 1 NEUROCAP Research Group, Psychology Department and Human Neuroscience Laboratory, Faculty of Psychology and Education, Universidad Loyola Andalucía, Avenida de las Universidades, 2, 41704 Dos Hermanas, Spain; slrodriguez@uloyola.es (S.L.-R.); bpostigo@uloyola.es (B.P.-A.); 2 IRENEA-Instituto de Rehabilitación Neurológica, C/ de Callosa d’en Sarrià, 12, Jesús, 46007 Valencia, Spain; myrtha@irenea.es (M.O.-R.); marta.gomez@neurorhb.com (M.G.-H.); loles.navarro@irenea.es (M.D.N.-P.); joan.ferri@irenea.es (J.F.-C.); enoe@comv.es (E.N.-S.); 3 Experimental Psychology Department, Faculty of Psychology, University of Seville, 41018 Seville, Spain

**Keywords:** Auricular Vagal Neuromodulation Therapy (AVNT), Disorders of Consciousness (DoCs), Mismatch Negativity (MMN), Coma Recovery Scale Revised (CRS-R), Minimally Conscious State (MCS), Unresponsive Wakefulness Syndrome (UWS)

## Abstract

**Highlights:**

**What are the main findings?**

- Between-session MMN amplitude changes differed between the clinical control and healthy groups in the parieto-occipital region, with no significant differences between the two clinical groups.

**What are the implications of the main findings?**

- These findings suggest that AVNT may represent a potential adjunctive intervention for supporting the preservation of auditory deviance processing, as reflected by MMN responses.

**Simple Summary:**

Severe brain injury can cause lasting impairments of consciousness, creating major challenges for rehabilitation. We examined whether electrical stimulation applied to the outer ear, intended to activate the vagus nerve, was associated with changes in the brain’s automatic response to unexpected sounds. We recorded brain activity and assessed behavioral responses twice, comparing assessments with and without stimulation and using healthy volunteers as a reference. Changes in auditory brain responses differed between the clinical control group and healthy volunteers, but no significant differences in these changes were detected between the two clinical groups. These findings may help guide research on how the brain processes sounds during rehabilitation, but they do not establish a direct benefit of stimulation. Larger, carefully controlled studies are needed to clarify its potential role in recovery.

**Abstract:**

**Background/Objectives:** Auricular Vagal Neuromodulation Therapy (AVNT) constitutes a promising non-invasive therapeutic approach for Disorders of Consciousness (DoCs). However, evidence regarding its effectiveness remains limited. In this context, although the Mismatch Negativity (MMN) is a well-established neurophysiological index associated with recovery in DoCs, its modulation by AVNT has not yet been investigated. This study aimed to evaluate the effect of AVNT on MMN in DoCs. **Methods:** Forty-four participants with DoCs were evenly assigned to either an experimental or a control group, alongside twenty-two healthy participants used to establish normative data in neurotypical conditions. The latency, peak and peak-to-peak MMN amplitude were assessed as a complement to the Coma Recovery Scale Revised (CRS-R). Subsequently, only the experimental group received AVNT, followed by a second assessment across all groups. **Results:** The control group showed extensive changes across sessions in both peak and peak-to-peak amplitudes. In contrast, the experimental group exhibited a significant change only in peak amplitude for the fronto-central region, with no changes in peak-to-peak amplitude. Between-session changes in peak and peak-to-peak amplitudes differed between the control and healthy groups in the parieto-occipital region. After adjustment for the inter-session interval, the ANCOVA showed an overall group effect on peak-to-peak amplitude changes in the parieto-occipital and right regions. No differences were detected between the control and experimental groups or between the experimental and healthy groups. Finally, no significant effects were found for MMN latency. **Conclusions:** These findings could be compatible with a possible effect of AVNT, although the evidence remained insufficient to demonstrate a direct effect of the intervention.

## 1. Introduction

Disorders of Consciousness (DoCs) are disabling conditions that can arise from acquired brain injuries, such as Traumatic Brain Injury (TBI) or Non-Traumatic Brain Injury (No-TBI) [1]. Clinically, DoCs are classified as Unresponsive Wakefulness Syndrome (UWS) or Minimally Conscious State (MCS), based on behavioral assessments using the Coma Recovery Scale-Revised (CRS-R) [2].

Non-invasive neuromodulation techniques have been explored as potential therapeutic options for DoCs, but most lack strong clinical evidence to support their efficacy [3]. Among these, notable examples include repetitive transcranial magnetic stimulation (rTMS) and transcranial direct current stimulation (tDCS) [4], with the latter receiving Class II evidence for intervention [5,6].

Auricular Vagal Neuromodulation Therapy (AVNT), also referred to as transauricular vagus nerve stimulation, has recently gained interest as a non-invasive therapeutic approach for DoCs [7]. Anatomical studies have demonstrated that the auricular branch of the vagus nerve innervates the tragus and cymba of the ear, providing a biological rationale for targeted electrical stimulation [8]. Accordingly, non-invasive electrical stimulation of these auricular regions has been proposed to modulate both norepinephrine and serotonergic pathways via the vagus nerve. This modulation may affect multiple brain networks, including the ascending reticular activating system, the salience network, and the default mode network. Moreover, considering the anatomical substrates intrinsically involved in maintaining consciousness [9], AVNT may represent a particularly promising neuromodulatory approach for DoCs. However, despite some studies indicating improvements in total CRS-R scores following AVNT [10,11], these outcomes remain inconsistent across different studies [12]. Notably, recovery in DoCs is characterized by marked variability and spontaneous fluctuations over time. As a result, behavioral assessments such as the CRS-R, while clinically indispensable, may be insensitive to subtle or early neural changes, underscoring the need for objective neurophysiological markers beyond overt behavior.

Although AVNT shows potential to enhance behavioral recovery of consciousness, the underlying neurophysiological mechanisms remain poorly understood. Previous studies have examined AVNT-related changes in Mismatch Negativity (MMN) in other clinical populations [13,14], and preliminary findings in DoC cohorts have been reported in a conference abstract [15]. Given this background, the present study provides a comprehensive analysis of MMN changes following AVNT in this population. Notably, this event-related potential component has been shown to possess prognostic value in predicting recovery of consciousness [16] and may be used as a potential marker of functional modifications after AVNT. In fact, MMN has been considered a well-established marker of auditory sensory function [17], being proposed to discriminate between patients who regained consciousness or not [18]. Furthermore, a positive correlation has been reported between the absolute increase in MMN amplitude in fronto-central regions and total CRS-R scores [19], which may reflect the recovery of behavioral responses to simple commands and an improved capacity for environmental interaction in patients [20].

Given the limited research on how AVNT modulates MMN in DoCs and the acknowledged need for multimodal assessments [21,22], the current study aimed to investigate these effects through a two-session electroencephalographic protocol, compared to neurotypical conditions. Forty-four participants with DoCs were equally distributed to a control group or an experimental group, while twenty-two neurotypical volunteers served as a healthy group. All participants underwent an initial recording during a passive two-tone auditory oddball paradigm to elicit the MMN component. Following this session, only the experimental group received forty sessions of AVNT over approximately thirty days. All participants then underwent a second electroencephalographic recording session. For clinical groups, CRS-R was also assessed concurrently with the recordings. The MMN peak amplitude and latency, along with CRS-R scores, were analyzed across groups, clinical diagnoses, and etiologies, comparing both sessions. Although previous studies conducted by the research group reported an increase in total CRS-R scores across sessions in both the experimental and control groups [23], the present study aimed to determine whether AVNT produced changes in MMN responses that differed from those observed in the control group. Specifically, it was hypothesized that only the experimental group would exhibit a shift toward more normative MMN characteristics following AVNT, potentially reflecting enhanced auditory discrimination, in line with findings reported in other clinical conditions, such as tinnitus [13]. Finally, this approach highlights the value of neurophysiological measures for guiding and evaluating novel therapeutic interventions in Disorders of Consciousness.

## 2. Materials and Methods

The protocol employed in the present study followed a methodological framework previously described for AVNT combined with electroencephalographic assessment in disorders of consciousness [24].

### 2.1. Ethical Statement

This study was approved by the Clinical Research Ethics Committee of the Hospital Clínic Universitari de València (No. 2019/002, 28 February 2019) and the Clinical Research Ethics Committee Sevilla Sur of the Hospital Universitario Virgen de Valme (No. 0271-N-23, 27 February 2024). The study also adhered to the principles of the Declaration of Helsinki (1964) and its subsequent amendments. 

Informed consent was obtained from healthy participants and from the legal representatives of all participants with disorders of consciousness. For minors, consent was obtained from their parents or legal guardians.

### 2.2. Participants

Two categories of participants were established in the clinical sample: an experimental group and a control group. These groups were further subdivided by diagnosis (MCS or UWS) and etiology (TBI or No-TBI). In addition, a healthy group was included to control for non-specific effects associated with the two-session design, such as habituation, and to provide a neurotypical reference framework.

The approved study protocol did not establish predefined age limits. Suitability for AVNT was assessed individually by the medical team based on clinical status and the absence of contraindications. Exclusion criteria included the presence of cardiovascular, metabolic, or autonomic disorders, uncontrolled epilepsy, psychiatric conditions, metallic implants, pregnancy, or the use of medications that affect autonomic function [25].

The experimental group consisted of twenty-two participants diagnosed with disorders of consciousness, at least two months post-injury [26], who underwent forty sessions of AVNT treatment (see Section 2.4). All participants were required to have a stable CRS-R score for at least four consecutive weeks. This criterion was established to minimize the influence of spontaneous fluctuations and to increase the likelihood that subsequent neurophysiological changes reflected effects associated with AVNT stimulation rather than short-term variations in clinical status. Their mean age ($\bar{x}$ ± SD) was 40 ± 17 years, and the mean post-injury duration ($\bar{x}$ ± SD) was 9 ± 4 months.

Similarly, the control group presented similar characteristics to the experimental group, with all participants diagnosed with disorders of consciousness. However, a stable CRS-R score was not required in this case, as the aim was to characterize the spontaneous variability in clinical measures observed in this population in the absence of AVNT stimulation. In addition, participants in this group did not receive any active neuromodulation intervention or sham stimulation. A total of 22 participants were recruited, with a mean age ($\bar{x}$ ± SD) of 34 ± 17 years and a mean time since injury ($\bar{x}$ ± SD) of 6 ± 4 months.

The healthy group consisted of twenty-two neurotypical volunteers, with a mean age ($\bar{x}$ ± SD) of 33 ± 10 years.

Participants were not randomly assigned to the clinical groups. Recruitment was conducted on a rolling basis following admission to the rehabilitation unit, and AVNT was initiated progressively according to eligibility, patient availability, clinical and medical approval, absence of contraindications, and written informed consent from the legal representative. Consequently, the experimental group comprised assessment periods during which AVNT was administered, whereas the control group comprised assessment periods in which patients received usual care without AVNT.

The required sample size was determined a priori using G*Power 3.1.9.7 software (Heinrich-Heine-Universität Düsseldorf, Germany). The calculation was based on a mixed repeated measures ANOVA with three groups and two measures per group. A medium effect size (f = 0.25) was assumed, with a significance level of α = 0.05 and a statistical power of 1 − β = 0.95.

Demographic and clinical characteristics of all participants are presented in Table S1.

### 2.3. Timeframe of the Study

All participants underwent an initial electroencephalographic recording session during a passive auditory oddball paradigm, accompanied by an assessment using the Spanish version of the CRS-R in the clinical groups. Subsequently, only the experimental group received a total of forty sessions of AVNT (see Section 2.4). Finally, a second electroencephalographic session was carried out with all participants, again accompanied by CRS-R assessments in the clinical groups. The healthy group underwent the same two recording sessions as the other groups to control for test–retest effects and temporal variability.

The temporal details of the study are illustrated in Figure S1 and detailed in Table S2.

### 2.4. Auricular Vagal Neuromodulation Therapy

Participants in the experimental group received two daily thirty-minute sessions of AVNT (Parasym PK01 Device; Parasym Ltd., London, UK) for a total of forty sessions, applied to the tragus of the left ear. Sinusoidal stimulation was delivered at a current intensity of 1.5 mA, a pulse repetition rate of 20 Hz, and a pulse width of 250 µs. The stimulation parameters were selected based on the tolerance threshold reported in previous studies [11] and were applied uniformly to all participants without individual adjustment. In contrast, participants in the control and healthy groups did not receive any active neuromodulation or sham stimulation.

### 2.5. Routine Therapeutic Activities

Participants in the clinical groups continued their standard rehabilitation program, which included pharmacological management, as well as multidisciplinary and multisensory stimulation therapy delivered by the clinical team. These routine interventions followed each center’s established clinical guidelines and were not modified by study participation. In this context, all participants received 20 therapy sessions per week, including ten 1-h multisensory therapy sessions and ten 1-h physiotherapy sessions. Neither the content nor the scheduling of these therapies differed between the experimental and clinical control groups, and no routine intervention involved active neuromodulation.

### 2.6. Clinical Evaluation

Participants in both control and experimental clinical groups were assessed weekly using the Spanish version of the CRS-R [27], administered in a quiet, well-lit room free of distractions by experienced clinical personnel. More detailed descriptions of the clinical evaluation procedures and the CRS-R results for the study cohort have been previously reported by our research group [23].

### 2.7. Electroencephalographic Evaluation

Two electroencephalographic recording sessions were conducted in all participants to evaluate automatic auditory deviance processing using a controlled passive oddball paradigm. Additionally, general recommendations for the use of electroencephalographic techniques in disorders of consciousness were followed [28,29,30].

#### 2.7.1. Recording

The electroencephalographic signal of the participants was simultaneously acquired and amplified during the recording sessions using an Enobio 8 cap (Fz, Cz, Pz, Oz, C3, P3, C4, P4) from Neuroelectrics^®^ (Barcelona, Spain), connected via Bluetooth to the company’s dedicated software. Signals were externally referenced to the right earlobe, with impedance maintained below 10 kΩ and a sampling rate of 500 Hz.

The recording sessions were conducted in a dedicated room in the hospital with stable light and temperature conditions. The experimental setup consisted of a stimulus presentation computer connected to a recording computer with loudspeakers (Trust International B.V., Dordrecht, The Netherlands, 28W, 4 Ω impedance, 20–20,000 Hz frequency range). Clinical participants were positioned according to clinical recommendations, facing the speakers, either in their adapted wheelchair or in bed, at a distance of 70–80 cm. The presentation intensity (dB SPL) was not measured and no sound level calibration was performed. In addition, all procedures were conducted with careful respect for their dignity, ensuring clear communication and appropriate explanations prior to recording. Additionally, the arousal facilitation protocol was performed before the tasks to prevent participants from falling asleep, including clapping and calling them by name [31].

Participants then listened passively to an oddball paradigm [32] designed in E-Prime 2.0 (Psychology Software Tools, Pittsburgh, PA, USA), with no task requirements and minimizing distractions. In this case, 50 deviant high tones (1000 Hz) were randomly presented among 150 standard low tones (398 Hz), with an intertrial interval of 1500 ms, a configuration commonly used to elicit MMN and automatic auditory deviance detection [33]. High-frequency tones had a duration of 70 ms with 10-ms rise and fall times, whereas low-frequency tones had a duration of 30 ms with 5-ms rise and fall times.

#### 2.7.2. Data Processing

The electroencephalographic signals were processed using BrainVision Analyzer 2^®^ software (Brain Products GmbH, Gilching, Germany), following the protocol illustrated in Figure S2.

Recordings were visually inspected for defective channels. When needed, interpolation used fourth-order spherical splines, Legendre polynomials of maximum degree ten, and λ = 10^−5^ [34]. Recordings with more than one defective channel were excluded [35]; only Fz required interpolation, on two occasions. Signals were then downsampled to 250 Hz using BrainVision Analyzer’s default anti-aliasing filter [36], improving computational efficiency while retaining the 2–8.5 Hz frequency range relevant to MMN analysis [29,37].

Next, the recording was segmented into different epochs by taking the 200 ms prior to stimulus onset and the following 1000 ms, separately for each deviant or standard stimulus type [38]. Additionally, baseline correction was applied using the pre-stimulus interval.

Once the recording was segmented, a 50 Hz notch filter was applied to remove artifacts caused by the European mains supply. Subsequently, two eighth-order infinite impulse response (IIR) filters were applied: a high-pass filter at 0.5 Hz and a low-pass filter at 30 Hz, to suppress frequencies outside the range of interest [39]. Filtering was performed independently in three sequential phases to avoid signal distortions due to filter overlaps. Subsequently, artifacts were visually identified and defective segments were semi-automatically removed using a voltage threshold of ±80 µV [40], discarding intervals with voltages below 0.5 µV for more than 100 ms. Due to the limited number of electrodes and the absence of dedicated ocular channels, independent component analysis (ICA) was not applied.

Only recordings with more than twenty valid segments were included in the signal analysis, following recommendations for the study of event-related components [41]. Details on the number of retained epochs for each group and condition can be found in Table S3. Subsequently, all valid segments in each recording were averaged separately for each deviant and standard tone, in order to enhance the genuine brain activity elicited by the stimulus and smooth out non-specific activity [42].

Then, the signal obtained by averaging the standard-stimuli trials was subtracted from the signal corresponding to the deviant-stimuli trials to isolate neural activity specifically associated with stimulus novelty while minimizing nonspecific responses [30]. This subtraction yielded the conventional deviant-minus-standard difference waveform and did not constitute a normalization procedure. Subsequently, an eighth-order IIR low-pass filter with a 10 Hz cutoff and baseline correction over the first 200 ms were performed to smooth the signal and facilitate evoked potential detection, based on previous studies [39]. The MMN component was identified as the most negative global peak of the difference waveform within the 80–300 ms time window following stimulus onset, in the channel showing the most prominent MMN response [20,43]. If no peak was detected within this interval, only the immediately following negative deflection was considered if its morphology and spatial distribution across the available electrodes were compatible with the MMN. The same criteria were applied systematically, and visual inspection was used to verify peak identification. Additionally, the inflection point immediately preceding the detected component was also identified.

The latency obtained for the MMN component was used as a reference for all other channels, and the corresponding amplitude was extracted within a ±5 ms window. In addition, the difference between the MMN peak and its preceding inflection point was calculated using the peak-to-peak method [44]. This procedure aimed to characterize brain activity specifically associated with the generation of the component of interest following stimulus presentation, relative to preceding nonspecific neural activity.

Additionally, specific regions of interest (ROIs) were established by grouping channels into Fronto-Central (Fz + Cz), Parieto-Occipital (Pz + Oz), Left (C3 + P3), and Right (C4 + P4) [45], ensuring that each ROI comprised an equal number of adjacent electrodes and that no electrodes were shared between ROIs. This distribution helped reduce the risk of imbalance in statistical comparisons. At the same time, it allowed all brain regions to be considered, which is particularly important in DoCs, as they may present alterations in the canonical topographical distribution of the MMN. Likewise, topographic maps corresponding to each condition and group were obtained.

Finally, peak and peak-to-peak amplitude values were normalized based on the healthy group by calculating z-scores. For this purpose, the mean of the healthy group was subtracted from each participant’s individual value, and the result was divided by the standard deviation of the healthy group. Normalization was performed separately by sex, ROI, and session. Thus, each value expressed the participant’s deviation from the corresponding healthy-control reference distribution.

### 2.8. Statistical Analysis

#### 2.8.1. Demographic Characteristics of the Participants

A chi-square (χ^2^) test was performed to evaluate the distribution of sex across the different groups, as well as among diagnoses and etiologies within the clinical groups. Additionally, age and time since injury data were subjected to Shapiro–Wilk and Levene’s tests, to assess normality and homoscedasticity assumptions. Subsequently, a one-way ANOVA with Bonferroni post-hoc comparisons was conducted between groups or subgroups if the assumptions of normality and homoscedasticity were met. If these assumptions were not satisfied, the Kruskal–Wallis test was applied, followed by Mann–Whitney U post-hoc tests with Bonferroni correction.

Additionally, when numerical demographic variables differed significantly between the comparative groups, ANCOVAs were conducted as a sensitivity analysis. Groups were included as the between-subjects factor, the demographic variables as covariates, and inter-session changes in MMN amplitudes as the dependent variable. Similarly, all MMN variables were compared between male and female participants using Mann–Whitney U tests, with Bonferroni correction for multiple comparisons.

#### 2.8.2. Timeframe

Between-group differences in the inter-session interval were assessed using the procedures described in Section 2.8.1: one-way ANOVA when normality and homoscedasticity assumptions were met, or Kruskal–Wallis tests otherwise, followed by Bonferroni-adjusted post hoc comparisons.

Additionally, an ANCOVA was conducted as a sensitivity analysis. Group was included as the between-subjects factor, the inter-session interval as a covariate, and inter-session changes in MMN amplitude as the dependent variable. This analysis assessed whether the observed changes in MMN amplitude remained after controlling for differences in session spacing.

#### 2.8.3. Clinical Evaluation

For statistical analysis, the CRS-R score closest in time to each electroencephalographic session was selected, to ensure consistency across participants and to minimize day-to-day fluctuations.

Shapiro–Wilk and Levene’s tests were used to assess normality and homogeneity. When both assumptions were satisfied, an ANOVA followed by Bonferroni-adjusted post hoc comparisons was performed. When the assumptions were not met, within-group longitudinal differences were assessed using the Wilcoxon test. Between-group differences were evaluated using the Kruskal–Wallis test, followed by Mann–Whitney U post hoc comparisons with Bonferroni correction. Additionally, between-group differences in inter-session changes were assessed using the Mann–Whitney U test.

#### 2.8.4. Electroencephalographic Data

The statistical analysis of the electroencephalographic data collected in the study was performed using the Python 3.12.12 programming language and following recommendations for medical research with small sample sizes [46], as indicated in Figure S3.

First, outliers in the studied parameters were identified and excluded according to established guidelines. For this purpose, the Median Absolute Deviation (MAD) was computed for each region of interest and session within each group, as well as within diagnoses and etiologies for additional analyses. MAD was defined as the median of the absolute deviations from the group median, scaled by the factor b = 1.4826. Outliers were considered values outside the range of the median ±2.5 × MAD, following previous recommendations [47]. Records with outliers in more than two regions of interest during the same session were excluded from the analysis, leaving the participants listed in Table S4.

Subsequently, data normality was assessed using a Shapiro–Wilk test, considering the reduced sample size. Normality was assumed when *p* ≥ 0.05. Additionally, if the normality assumption was confirmed, a Levene’s test was performed to check homoscedasticity, assuming equal variance for *p* ≥ 0.05.

A mixed repeated-measures ANOVA was performed under the assumptions of normality and homoscedasticity to detect significant differences between groups. The values obtained in the different evaluations (first session and second session) for the four regions of interest (Fronto-Central, Parieto-Occipital, Left and Right) were considered as the within-subject factor, while the group (Healthy, Experimental and Control) was used as the between-subject factor. Subsequently, a two-tailed Bonferroni post-hoc analysis was conducted to identify possible significant differences between specific conditions. For this purpose, *p*-values were adjusted by multiplying them by the number of comparisons made, considering a significance threshold of *p*_Bonferroni_ < 0.05.

If the assumptions of normality or homoscedasticity were not met, non-parametric tests were applied instead. Thus, a two-tailed Wilcoxon test was first performed for each region of interest and group separately, to detect significant differences between both sessions. Likewise, to identify possible differences between groups within the same session, a Kruskal–Wallis test was applied, followed by post-hoc comparisons using the two-tailed Mann–Whitney U test with Bonferroni correction for three possible pairs. Next, a Friedman test was used to detect significant differences between regions of interest within the same group and session, accompanied by post-hoc comparisons using the two-tailed Wilcoxon test with Bonferroni correction for six possible pairs. Because the non-parametric approach did not allow testing the interaction between groups and sessions, the difference between sessions was calculated for each participant. These difference scores were then compared across groups using a Kruskal–Wallis test, followed by post hoc Mann–Whitney U tests with Bonferroni correction for multiple comparisons. In all cases, the significance threshold was maintained at *p* < 0.05 and compared with the adjusted *p*-value (*p*_Bonferroni_) obtained in the statistical test.

Additionally, Spearman correlation analyses were conducted to examine the relationship between session-to-session changes in MMN amplitudes and corresponding changes in CRS-R scores. A complementary Bonferroni correction was applied to account for multiple comparisons, considering the different groups and ROIs.

Complementary and exploratory analyses, similar to those previously described, were conducted incorporating diagnosis (MCS vs. UWS) or etiology (TBI vs. No-TBI) as between-subject factors.

Finally, effect size was calculated using Cohen’s d when the post-hoc analysis reached statistical significance [48], as well as effect size r.

### 2.9. Safety Considerations

To ensure participant safety during stimulation, the occurrence of adverse events was taken into consideration and participant discomfort was monitored clinically.

## 3. Results

### 3.1. Normality and Homoscedasticity Assumptions

The assumptions of normality and homoscedasticity could not be met for all comparative groups, according to the Shapiro–Wilk and Levene tests. Therefore, non-parametric tests were used in all primary comparative analyses. Nevertheless, ANCOVA analyses were performed as sensitivity analyses to account for potential confounding covariates.

### 3.2. Demographic Characteristics of the Participants

The healthy group had a higher proportion of women than the clinical groups [χ^2^(2, 66) = 9.80, *p* = 0.007]. Significant sex-related differences were observed in normalized peak amplitude in both sessions and in change scores. No significant sex differences were detected after correction for multiple comparisons in unnormalized measures or normalized peak-to-peak amplitude and latency. Diagnosis and etiology distributions did not differ significantly between clinical groups. Detailed results are provided in Supplementary Materials, Supplementary Section S4.

Age did not differ significantly across experimental, control, and healthy groups. Within the clinical groups, age also did not differ across diagnoses or etiologies. However, the experimental group showed a significantly longer time since injury compared to the control group [U = 340, *p* = 0.008, d = 0.901, r = 0.411], while no differences in these variables were found across the different diagnoses and etiologies. Importantly, ANCOVA analyses indicated that the time since injury did not have a significant effect on changes in MMN amplitude across sessions in any of the groups.

All demographic factors that could constitute potential confounding variables in the subsequent interpretation of the electroencephalographic results are presented in Table 1.

### 3.3. Timeframe

Regarding the time elapsed between the two electroencephalographic sessions, the Kruskal–Wallis test revealed significant differences among the three groups [H(2) = 7.76, *p* = 0.026]. Post-hoc analyses using the Mann-Whitney U test with Bonferroni correction showed that the healthy group had a significantly longer interval between sessions compared to the experimental group [U = 128, *p*_Bonferroni_ = 0.039, d = 0.877, r = 0.402]. No significant differences were observed between the control and experimental groups [U = 195, *p*_Bonferroni_ = 0.824, d = 0.396, r = 0.165] or between the control and healthy groups [U = 325, *p*_Bonferroni_ = 0.158, d = 0.730, r = 0.292].

However, the ANCOVA analyses did not reveal any significant effect of the inter-session interval on changes in MMN amplitude across sessions in any of the groups. After adjustment for the inter-session interval, the ANCOVA showed significant overall group effects on changes in peak-to-peak amplitude in the parieto-occipital and right regions, as reported in Section 3.4.3.

### 3.4. Classification by Groups

This section presents the results of MMN assessments based on normalized values, in comparison with the healthy group. Analyses using unnormalized values are available in Supplementary Section S3.1. It should be noted that the results are expressed as z-scores, following normalization based on the healthy group. In this context, positive and negative values indicate deviations above and below the normative pattern, respectively. However, these values should not be interpreted as directly reflecting the polarity of the MMN amplitude.

In addition, the unnormalized event-related potential waveform eliciting the MMN component, obtained by subtracting the standard stimuli from the deviant stimuli, is shown in Figure S4, while the waveforms for each individual condition are available in Figure S5.

#### 3.4.1. Clinical Evaluation

According to the main outcomes, no significant differences were detected between the experimental and control groups at baseline, neither at the second assessment nor in the change scores across sessions. In contrast, a non-parametric Wilcoxon test revealed a significant increase in total CRS-R scores across sessions in the experimental group [W(21) = 0, *p* = 0.003, d = 0.725, r = 0.630], given violations of normality and homoscedasticity assumptions. However, the control group also showed a pattern of improvement [W(21) = 0, *p* = 0.003, d = 0.659, r = 0.629].

The CRS-R scores are presented in Table 2 for the two clinical groups, including scores for each session and changes between sessions.

#### 3.4.2. Peak Amplitude

The control group showed a significant change from negative to positive values between sessions in the fronto-central [W(21) = 58, *p* = 0.025, d = 0.579, r = 0.474], parieto-occipital [W(21) = 50, *p* = 0.011, d = 0.552, r = 0.529], left [W(21) = 48, *p* = 0.009, d = 0.572, r = 0.543], and right [W(21) = 52, *p* = 0.014, d = 0.623, r = 0.516] regions. The experimental group also showed a significant shift, but only in the fronto-central region [W(21) = 62, *p* = 0.036, d = 0.521, r = 0.446].

No significant differences were detected among the three groups during the first session according to the Kruskal–Wallis test. In contrast, according to the Friedman test, the experimental group (EG) [Q(3) = 13.80, *p* = 0.003] showed more negative values in the fronto-central (FC) region than in the right region [W = 47, *p*_Bonferroni_ = 0.049, d = 0.238, r = 0.550]. No other significant topographical differences were observed in any group.

During the second session, however, the Kruskal–Wallis test reached significance in the fronto-central [H(2) = 10.41, *p* = 0.005], parieto-occipital [H(2) = 8.909, *p* = 0.012], left [H(2) = 11.87, *p* = 0.003], and right [H(2) = 10.50, *p* = 0.005] regions. Specifically, the control group showed significantly more positive values than the healthy group in the fronto-central [U = 359, *p*_Bonferroni_ = 0.006, d = 0.959, r = 0.474], parieto-occipital [U = 354, *p*_Bonferroni_ = 0.009, d = 1.022, r = 0.456], left [U = 373, *p*_Bonferroni_ = 0.002, d = 1.222, r = 0.526], and right [U = 376, *p*_Bonferroni_ = 0.001, d = 1.267, r = 0.537] regions. No significant differences were observed between the control and experimental groups, or between the experimental and healthy groups. In addition, no topographic differences were observed in any group during the second session.

Finally, the between-session difference scores revealed significant group differences in the parieto-occipital region, according to the Kruskal–Wallis test [H(2) = 6.12, *p* = 0.046]. Post-hoc comparisons indicated that the control group exhibited a greater between-session change than the healthy group [U = 331, *p*_Bonferroni_ = 0.047, d = 0.700, r = 0.370]. No significant differences were observed between the control and experimental groups or between the experimental and healthy groups.

No significant correlations between changes in CRS-R scores and MMN amplitudes were detected in any group or ROI.

**Figure 1:**
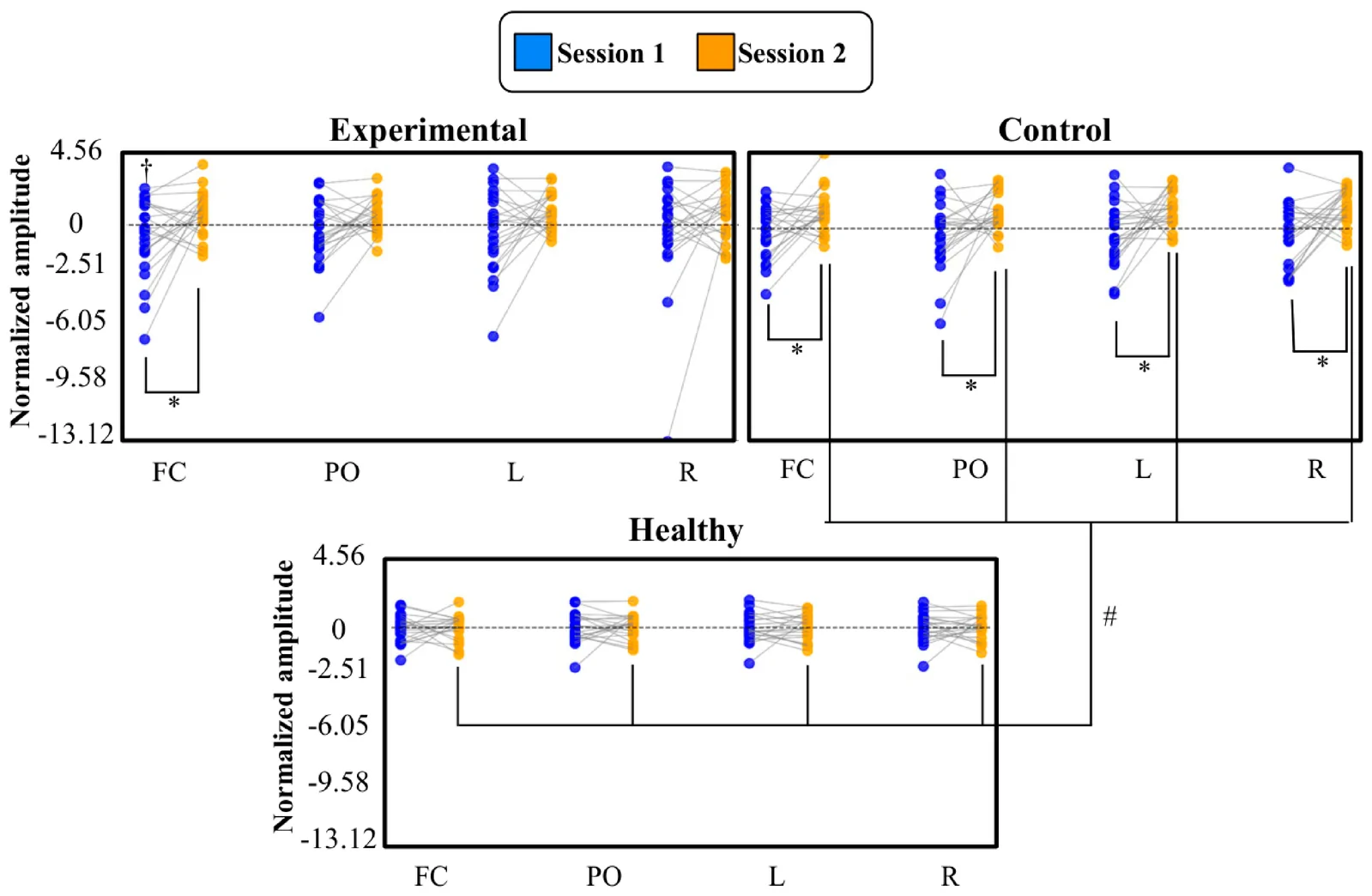
Distribution of peak MMN amplitudes across participants, normalized to the healthy group. Note: Values are z-score normalized relative to the healthy group and shown for the Fronto-Central (FC), Parieto-Occipital (PO), Left (L), and Right (R) regions. Data from both the first (blue) and second (orange) recording sessions are displayed. The asterisk symbol (*) represents significant differences between sessions (Wilcoxon test), whereas the hashtag symbol (#) indicates significant differences between groups (Kruskal–Wallis and Mann–Whitney U tests) and the dagger symbol (†) marks differences between regions (Friedman and Wilcoxon tests). The significance level for all tests was set at *p*_Bonferroni_ < 0.05.

**Figure 2:**
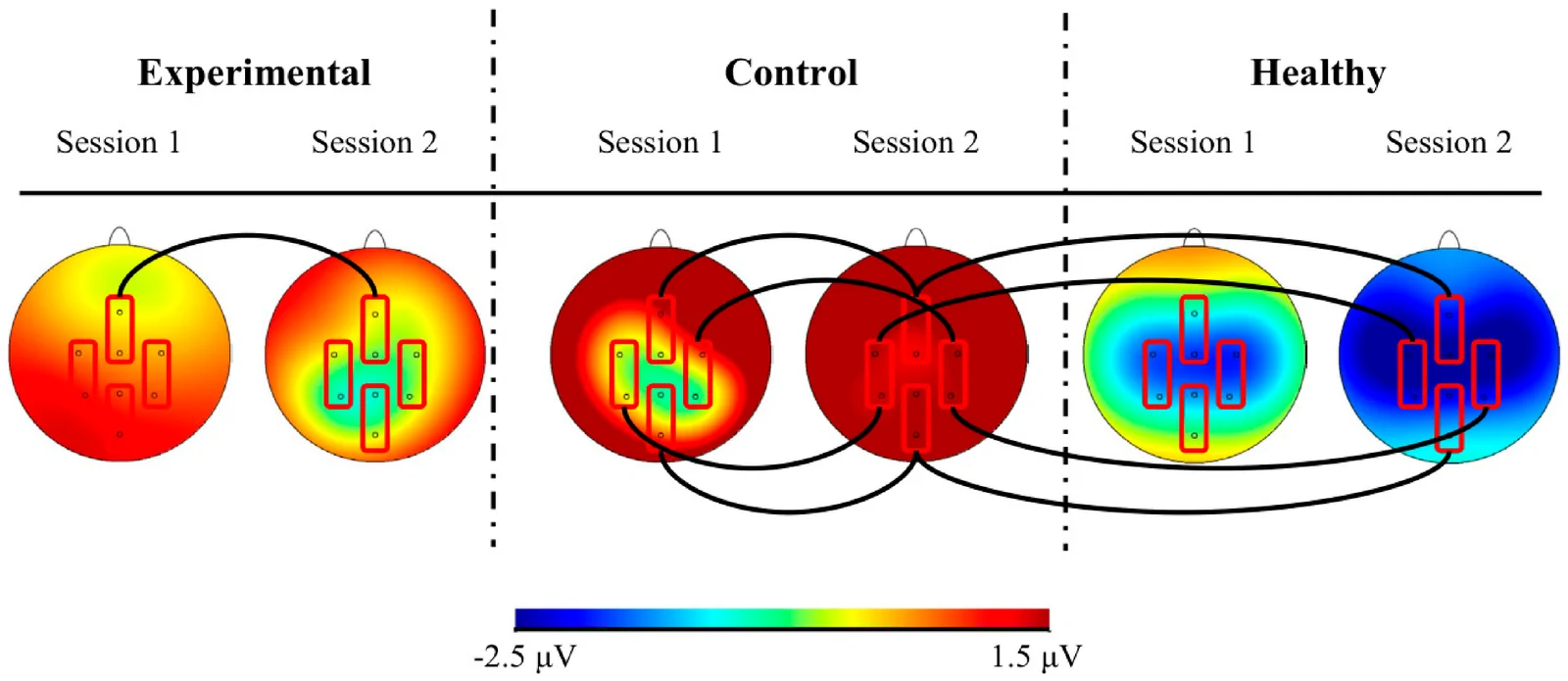
Topographic maps of unnormalized MMN peak amplitude by group and session. Note: Red rectangles indicate the different regions of interest (ROIs). In addition, black lines denote significant differences between ROIs across groups and sessions (*p*_Bonferroni_ < 0.05), where significance was determined using values normalized to the healthy group.

Table 3 presents peak amplitude values normalized relative to the healthy group, with the distribution of individual values shown in Figure 1 and unnormalized topographic distribution maps provided in Figure 2.

#### 3.4.3. Peak-to-Peak Amplitude

First, the control group showed a significant change from positive to negative values between sessions in the parieto-occipital [W(21) = 45, *p* = 0.007, d = 0.562, r = 0.564], left [W(21) = 55, *p* = 0.019, d = 0.615, r = 0.495], and right [W(21) = 44, *p* = 0.006, d = 0.677, r = 0.571] regions. No significant between-session differences were detected in the other groups.

No significant differences were detected among the three groups during the first session according to the Kruskal–Wallis test. However, according to the Friedman test, the control group [Q(3) = 18, *p* < 0.001] showed more positive values in the parieto-occipital region than the fronto-central [W = 21, *p*_Bonferroni_ = 0.001, d = 0.594, r = 0.730], left [W = 43, *p*_Bonferroni_ = 0.032, d = 0.428, r = 0.578], and right [W = 44, *p*_Bonferroni_ = 0.035, d = 0.520, r = 0.571] regions. No topographic differences were detected in other groups during the first session.

During the second session, the Kruskal–Wallis test reached significance for the right region [H(2) = 6.83, *p* = 0.033], where the control group showed more negative values than the healthy group [U = 127, *p*_Bonferroni_ = 0.036, d = 0.925, r = 0.385]. However, no significant differences were observed between the control and experimental groups, or between the experimental and healthy groups. The Friedman test was no longer significant, indicating that no topographical differences were detected for any group.

Finally, the analysis of between-session difference scores indicated significant group differences in the parieto-occipital region, as indicated by the Kruskal–Wallis test [H(2) = 6.12, *p* = 0.047]. Subsequent Mann–Whitney U tests revealed that the control group exhibited a greater change over time than the healthy group [U = 130, *p*_Bonferroni_ = 0.027, d = 0.780, r = 0.396]. No significant differences were observed between the control and experimental groups or between the experimental and healthy groups. Moreover, after adjustment for the interval between sessions, the ANCOVA showed a significant group effect in the parieto-occipital [F(2,61) = 3.24, *p* = 0.046] and right [F(2,61) = 3.79, *p* = 0.028] regions.

Uncorrected Spearman correlation analysis revealed a significant positive correlation between session-to-session changes in CRS-R scores and MMN peak-to-peak amplitude in the left region in the experimental group [Spearman’s rho = 0.439, *p* = 0.041]. However, this correlation did not remain significant after adjustment for multiple comparisons. In contrast, no significant correlation was observed in the control group.

**Figure 3:**
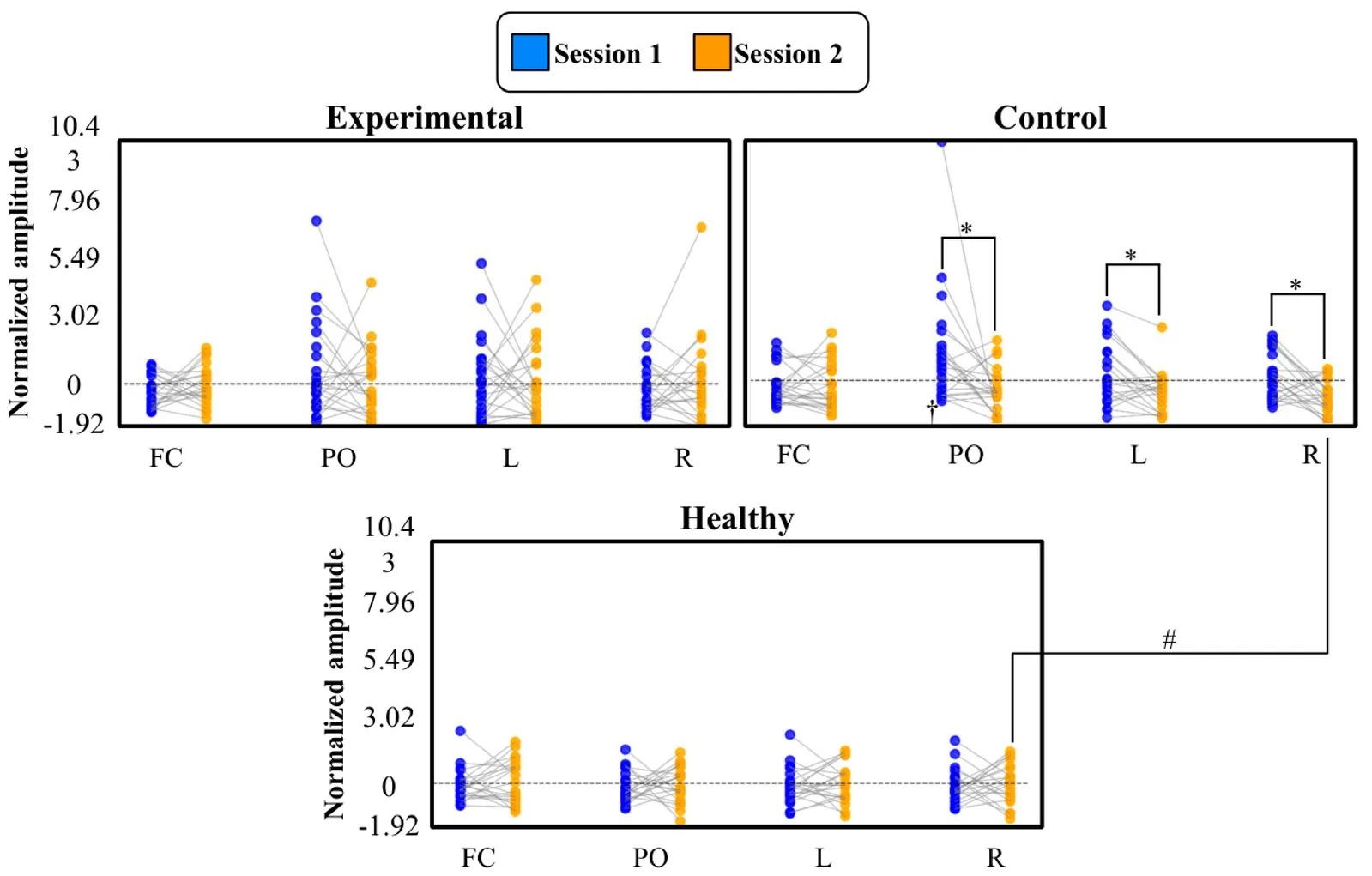
Distribution of peak-to-peak MMN amplitudes across participants, normalized to the healthy group. Note: Values are z-score normalized relative to the healthy group and shown for the Fronto-Central (FC), Parieto-Occipital (PO), Left (L), and Right (R) regions. Data from both the first (blue) and second (orange) recording sessions are displayed. The asterisk symbol (*) represents significant differences between sessions (Wilcoxon test), whereas the hashtag symbol (#) indicates significant differences between groups (Kruskal–Wallis and Mann–Whitney U tests) and the dagger symbol (†) marks differences between regions (Friedman and Wilcoxon tests). The significance level for all tests was set at *p*_Bonferroni_ < 0.05.

Normalized peak-to-peak amplitude values by group are presented in Table 4, with individual distribution plots shown in Figure 3.

### 3.5. Classification by Diagnoses

This section presents the results of MMN assessments based on normalized values, in comparison with the healthy group. Results using unnormalized values are available in Supplementary Section S3.2.

#### 3.5.1. Clinical Evaluation

Participants diagnosed with MCS exhibited higher total CRS-R scores than those with UWS in both sessions, independent of group assignment and consistent with their diagnostic status. Additionally, participants diagnosed with MCS in both the control [W = 0, *p*_Bonferroni_ = 0.031, d = 0.932, r = 0.718] and experimental [W = 0, *p*_Bonferroni_ = 0.011, d = 0.814, r = 0.656] groups showed an increase in total CRS-R scores across sessions, as indicated by the Wilcoxon test. However, no significant between-group differences were detected in the inter-session changes.

The CRS-R scores are presented in Table 5 for all diagnostic groups, including scores for each session and changes between sessions.

#### 3.5.2. Peak Amplitude

Significant between-session differences were detected for the UWS diagnosis within the control group, with values shifting from negative to positive in the fronto-central [W(12) = 12, *p* = 0.017, d = 0.849, r = 0.649], parieto-occipital [W(12) = 13, *p* = 0.021, d = 0.621, r = 0.630], left [W(12) = 16, *p* = 0.040, d = 0.650, r = 0.572], and right [W(12) = 10, *p* = 0.010, d = 0.858, r = 0.688] regions. A similar effect was also observed in the experimental group with MCS diagnosis, although only in the FC region [W(14) = 24, *p* = 0.041, d = 0.578, r = 0.530].

In contrast, no between-group differences were found for either session according to the Kruskal–Wallis test.

However, the Friedman test revealed significant topographical differences in the control group with MCS diagnosis only during the second session [Q(3) = 9.67, *p* = 0.021], where the right region showed more positive values than the fronto-central region [W = 0, *p*_Bonferroni_ = 0.023, d = 0.887, r = 0.888]. No other topographic differences were detected in any group.

No significant differences among diagnostic groups were found in the between-session difference score analyses, and no significant correlations with the CRS-R were observed.

**Figure 4:**
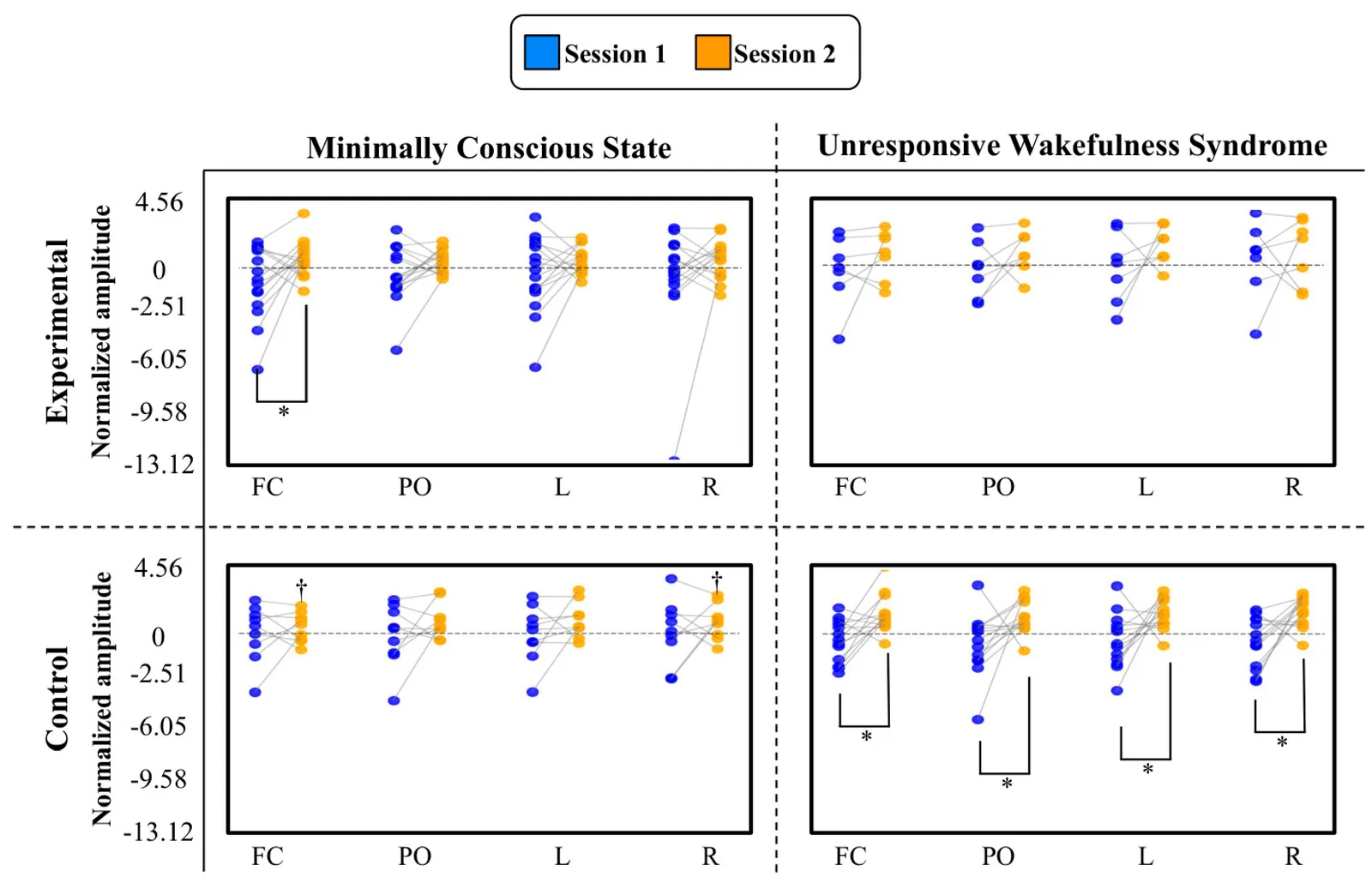
Distribution of peak MMN amplitudes across participants by diagnosis, normalized to the healthy group. Note: Values are z-score normalized relative to the healthy group and shown for the Fronto-Central (FC), Parieto-Occipital (PO), Left (L), and Right (R) regions. Data from both the first (blue) and second (orange) recording sessions are displayed. The asterisk symbol (*) represents significant differences between sessions (Wilcoxon test), and the dagger symbol (†) marks differences between regions (Friedman and Wilcoxon tests). The significance level for all tests was set at *p*_Bonferroni_ < 0.05.

**Figure 5:**
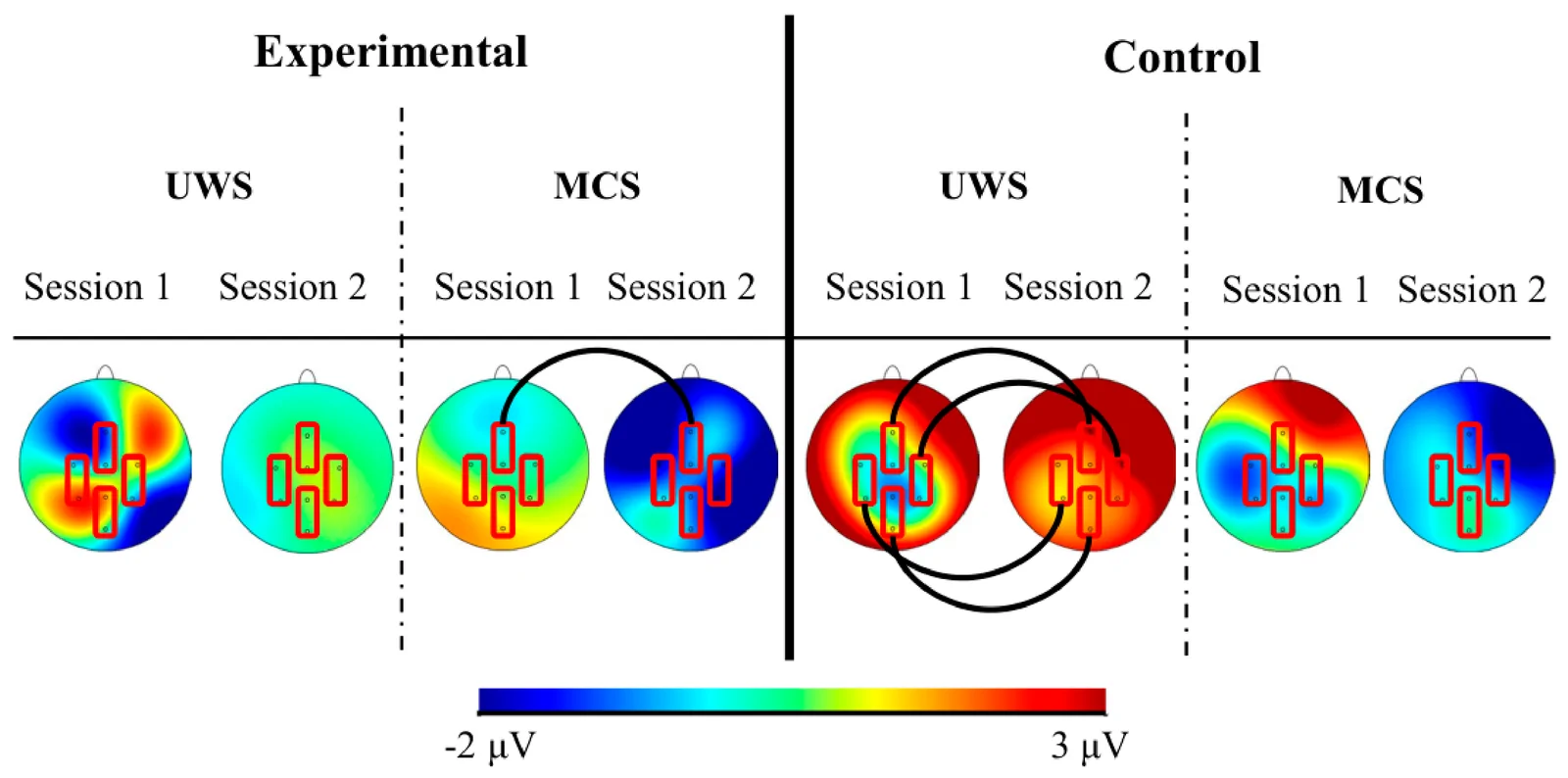
Topographic maps of unnormalized MMN peak amplitude by diagnoses and session. Note: Red rectangles indicate the different regions of interest (ROIs). In addition, black lines denote significant differences between ROIs across groups and sessions (*p*_Bonferroni_ < 0.05), where significance was determined using values normalized to the healthy group.

Table 6 presents values normalized to the healthy group, with individual distribution shown in Figure 4, along with unnormalized topographic distribution maps in Figure 5.

#### 3.5.3. Peak-to-Peak Amplitude

A significant between-session change was detected in the control group with UWS diagnosis, with values shifting from positive to negative in the parieto-occipital region [W(12) = 16, *p* = 0.040, d = 0.594, r = 0.572] and the left region [W(12) = 16, *p* = 0.040, d = 0.706, r = 0.572]. No significant between-session differences were observed in the other diagnostic groups.

The Kruskal–Wallis test did not detect significant differences between diagnostic groups during the first session. In contrast, the Friedman test revealed significant topographical differences in the control group with UWS diagnosis [Q(3) = 19.61, *p* < 0.001], with the parieto-occipital region showing higher positive values than the fronto-central [W = 2, *p*_Bonferroni_ = 0.004, d = 0.674, r = 0.843], left [W = 8, *p*_Bonferroni_ = 0.037, d = 0.503, r = 0.727], and right [W = 9, *p*_Bonferroni_ = 0.048, d = 0.647, r = 0.707] regions. No topographic differences were detected in other groups during the first session.

Neither the Kruskal–Wallis test nor the Friedman test yielded significant results during the second session, indicating that no significant differences were detected between groups or across topographical locations.

No significant differences among the diagnostic groups were found in the analyses of between-session difference scores.

Uncorrected Spearman correlation analysis revealed a significant positive correlation between session-to-session changes in CRS-R scores and MMN peak-to-peak amplitude in the right region in the experimental group with UWS diagnosis [Spearman’s rho = 0.774, *p* = 0.041]. However, this correlation did not remain significant after adjustment for multiple comparisons. In contrast, no significant correlation was observed in the control group.

**Figure 6:**
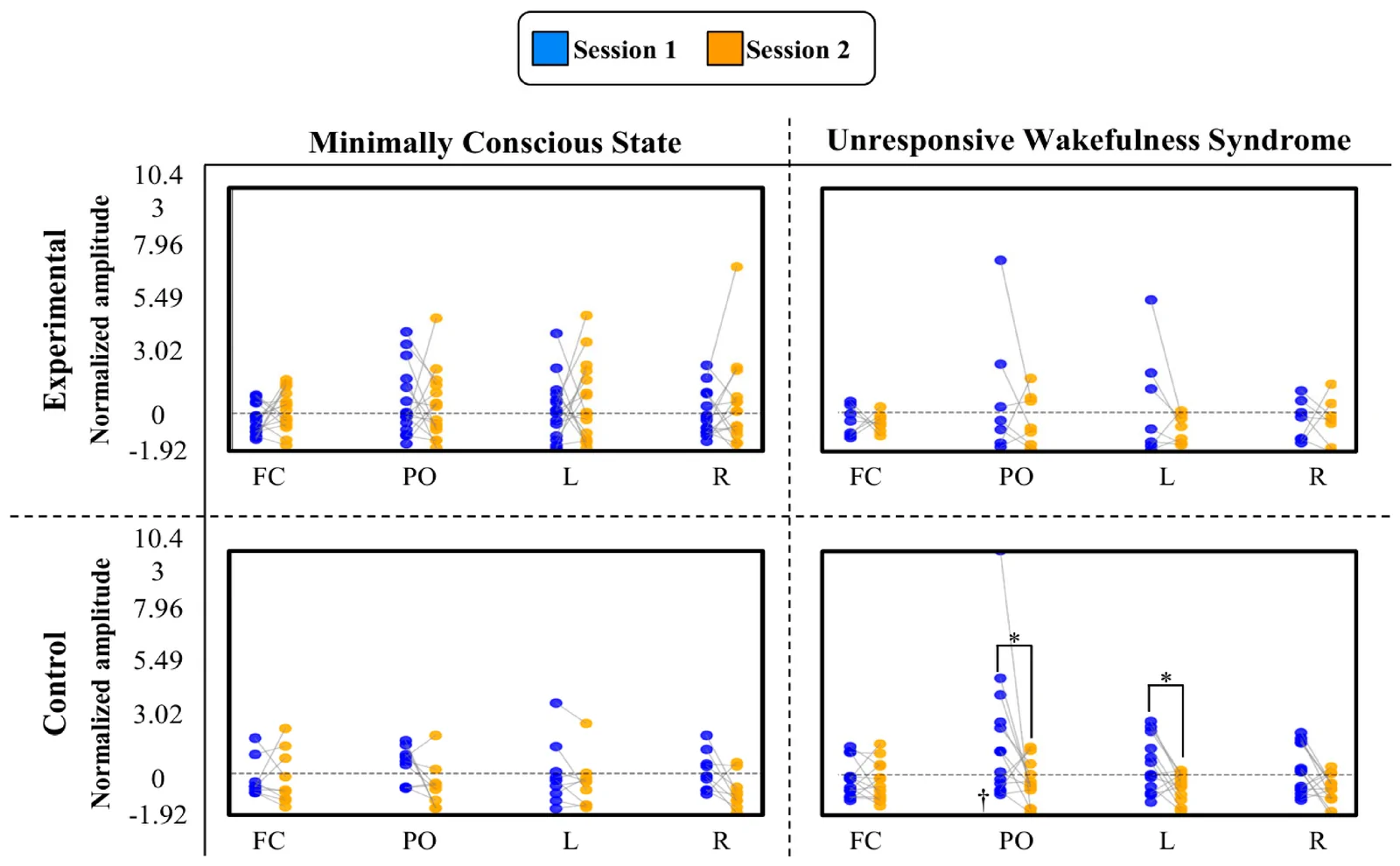
Distribution of peak-to-peak MMN amplitudes across participants by diagnosis, normalized to the healthy group. Note: Values are z-score normalized relative to the healthy group and shown for the Fronto-Central (FC), Parieto-Occipital (PO), Left (L), and Right (R) regions. Data from both the first (blue) and second (orange) recording sessions are displayed. The asterisk symbol (*) represents significant differences between sessions (Wilcoxon test), and the dagger symbol (†) marks differences between regions (Friedman and Wilcoxon tests). The significance level for all tests was set at *p*_Bonferroni_ < 0.05.

Normalized peak-to-peak amplitude values by diagnosis are presented in Table 7, with individual distribution plots shown in Figure 6.

### 3.6. Classification by Etiologies

This section presents the results of MMN assessments based on normalized values, in comparison with the healthy group. Results using unnormalized values are available in Supplementary Section S3.3.

#### 3.6.1. Clinical Evaluation

According to the Wilcoxon test, participants with TBI exhibited an increase in total CRS-R scores during the second session in both the control [W = 0, *p*_Bonferroni_ = 0.016, d = 0.697, r = 0.671] and experimental [W = 0, *p*_Bonferroni_ = 0.008, d = 0.849, r = 0.738] groups. However, no significant differences between etiologies were observed in either session or in the inter-session changes.

The CRS-R scores are presented in Table 8 for all etiological groups, including scores for each session and changes between sessions.

#### 3.6.2. Peak Amplitude

Only the control group with TBI etiology showed a change between sessions, shifting from negative to positive values in the second session for the fronto-central [W(12) = 14, *p* = 0.027, d = 0.716, r = 0.611] and left [W(12) = 12, *p* = 0.017, d = 0.680, r = 0.649] regions. No other between-session differences were detected in the remaining groups.

Although no significant differences between groups were found during the first session, the experimental group with TBI etiology [Q(3) = 13.20, *p* = 0.004] showed more positive values in the right region than in fronto-central [W = 0, *p*_Bonferroni_ = 0.003, d = 1.090, r = 0.883].

No differences between groups were found during the second session either. However, topographical differences were only observed in the experimental group with non-TBI etiology [Q(3) = 8.87, *p* = 0.031], where the left region showed more positive values than the fronto-central region [W = 0, *p*_Bonferroni_ = 0.023, d = 0.783, r = 0.889].

Finally, no significant differences among the etiological groups were found in the analyses of between-session difference scores, and no significant correlations with the CRS-R were observed.

**Figure 7:**
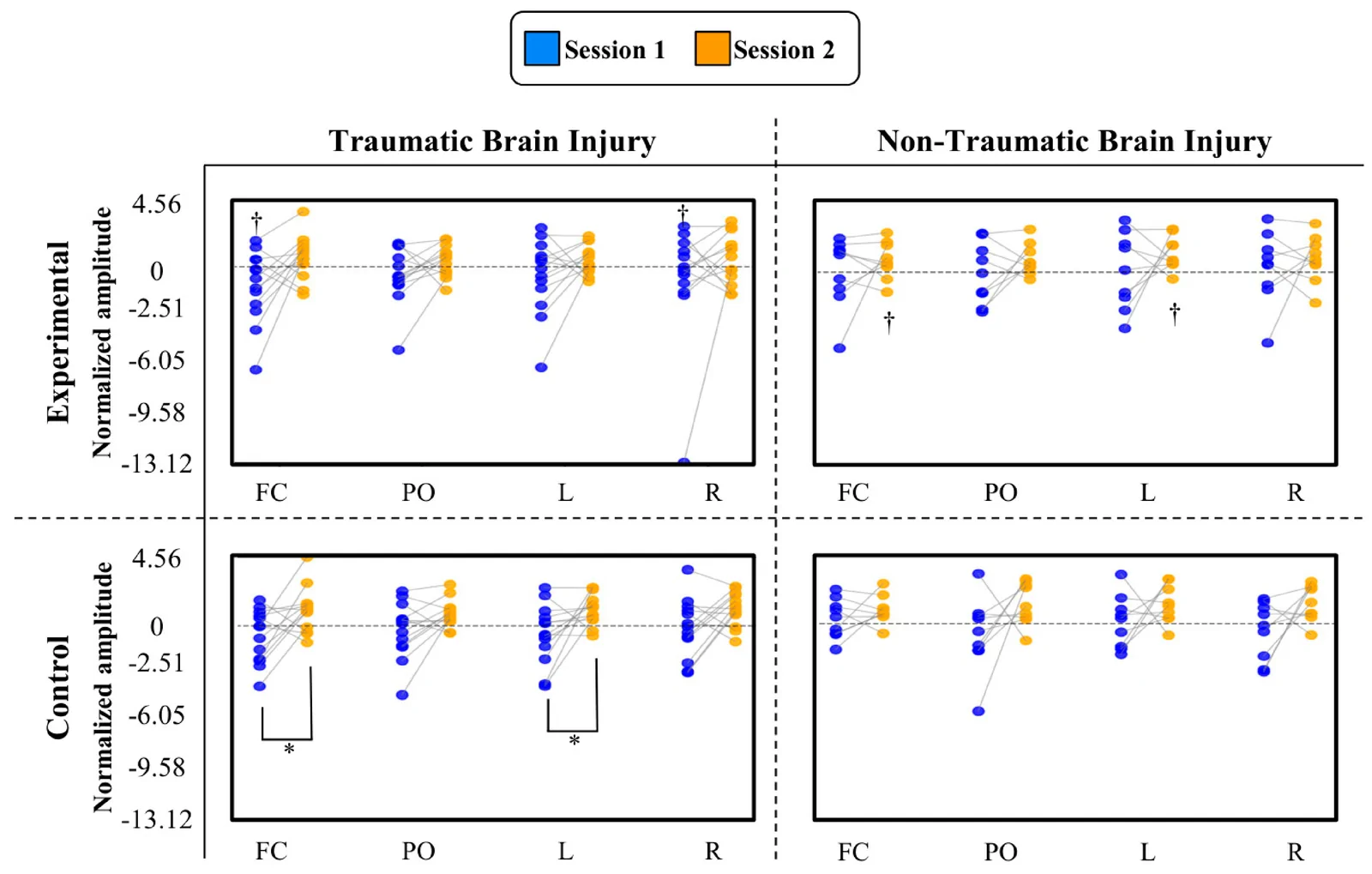
Distribution of peak MMN amplitudes across participants by etiologies, normalized to the healthy group. Note: Values are z-score normalized relative to the healthy group and shown for the Fronto-Central (FC), Parieto-Occipital (PO), Left (L), and Right (R) regions. Data from both the first (blue) and second (orange) recording sessions are displayed. The asterisk symbol (*) represents significant differences between sessions (Wilcoxon test), and the dagger symbol (†) marks differences between regions (Friedman and Wilcoxon tests). The significance level for all tests was set at *p*_Bonferroni_ < 0.05.

**Figure 8:**
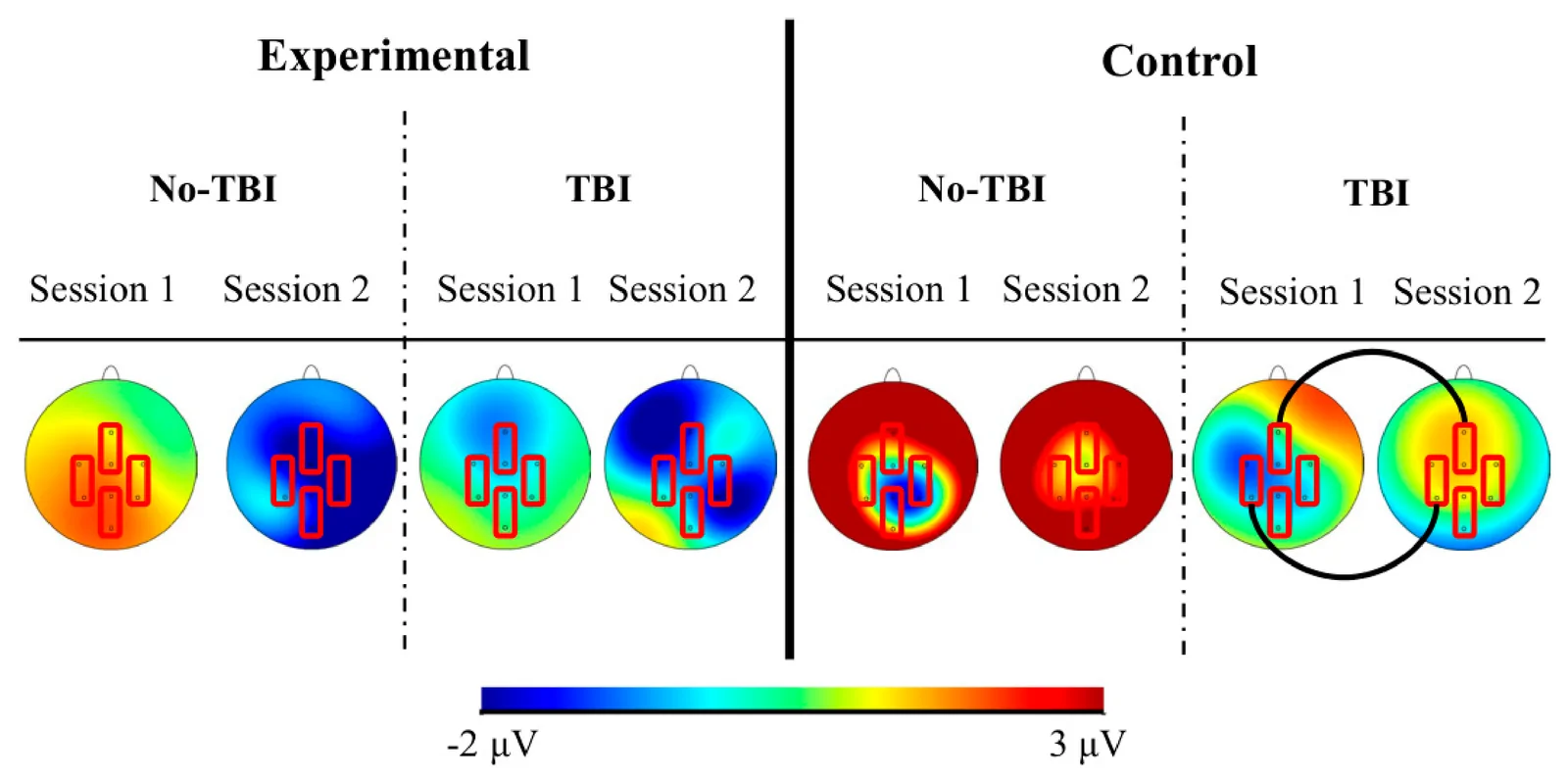
Topographic maps of unnormalized MMN peak amplitude by etiologies and session. Note: Red rectangles indicate the different regions of interest (ROIs). In addition, black lines denote significant differences between ROIs across groups and sessions (*p*_Bonferroni_ < 0.05), where significance was determined using values normalized to the healthy group.

Table 9 presents values normalized to the healthy group, with individual distribution shown in Figure 7, along with unnormalized topographic distribution maps in Figure 8.

#### 3.6.3. Peak-to-Peak Amplitude

First, control group with TBI etiology showed more negative values in the parieto-occipital [W(12) = 17, *p* = 0.048, d = 0.659, r = 0.552], left [W(12) = 16, *p* = 0.040, d = 0.652, r = 0.571], and right [W(12) = 11, *p* = 0.013, d = 0.815, r = 0.669] regions during the second session compared with the first session. No significant between-session differences were observed in the other etiological groups.

During the first session, although the Kruskal–Wallis test did not detect significant differences among the groups, the control group with non-TBI etiology [Q(3) = 13.93, *p* = 0.003] showed more positive values in the parieto-occipital region than in the fronto-central [W = 0, *p*_Bonferroni_ = 0.004, d = 0.685, r = 0.889] and the right regions [W = 1, *p*_Bonferroni_ = 0.047, d = 0.671, r = 0.849]. No other topographical differences were observed in the other groups.

During the second session, the Kruskal–Wallis test revealed significant differences in the right region [H(3) = 10.66, *p* = 0.014], where the control group with TBI etiology showed more negative values than the experimental group [U = 143, *p*_Bonferroni_ = 0.018, d = 0.969, r = 0.588]. No significant topographical differences were detected in this case according to the Friedman test.

Finally, no significant differences among the etiological groups were found in the analyses of between-session difference scores.

Uncorrected Spearman correlation analysis revealed a significant positive correlation between session-to-session changes in CRS-R scores and MMN peak-to-peak amplitude in the left region in the experimental group with TBI etiology [Spearman’s rho = 0.629, *p* = 0.021]. However, this correlation did not remain significant after adjustment for multiple comparisons. In contrast, no significant correlation was observed in the control group.

**Figure 9:**
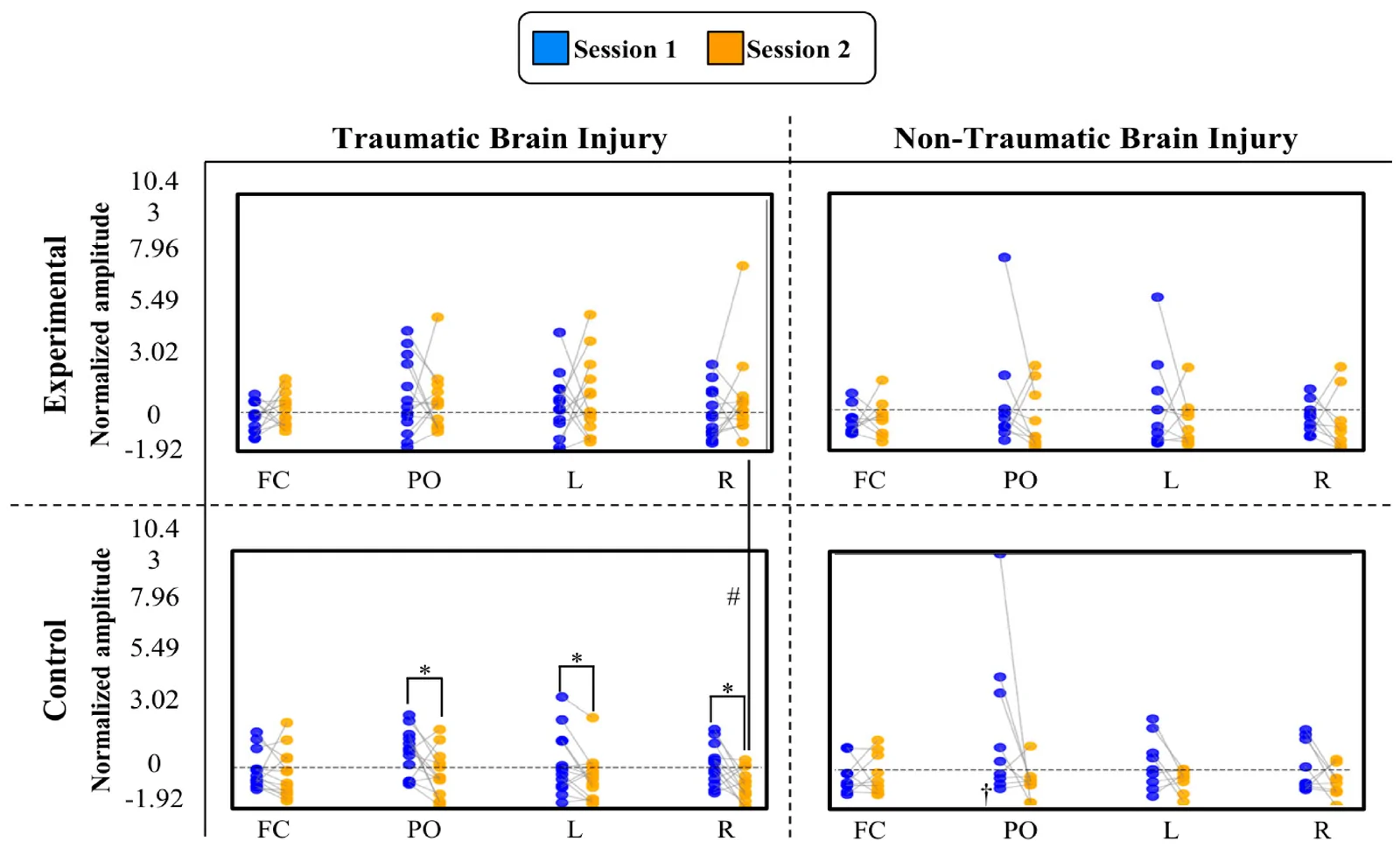
Distribution of peak-to-peak MMN amplitudes across participants by etiologies, normalized to the healthy group. Note: Values are z-score normalized relative to the healthy group and shown for the Fronto-Central (FC), Parieto-Occipital (PO), Left (L), and Right (R) regions. Data from both the first (blue) and second (orange) recording sessions are displayed. The asterisk symbol (*) represents significant differences between sessions (Wilcoxon test), whereas the hashtag symbol (#) indicates significant differences between groups (Kruskal–Wallis and Mann–Whitney U tests) and the dagger symbol (†) marks differences between regions (Friedman and Wilcoxon tests). The significance level for all tests was set at *p*_Bonferroni_ < 0.05.

Normalized peak-to-peak amplitude values by etiologies are presented in Table 10, with individual distribution plots shown in Figure 9.

### 3.7. Latency Analysis

**Figure 10:**
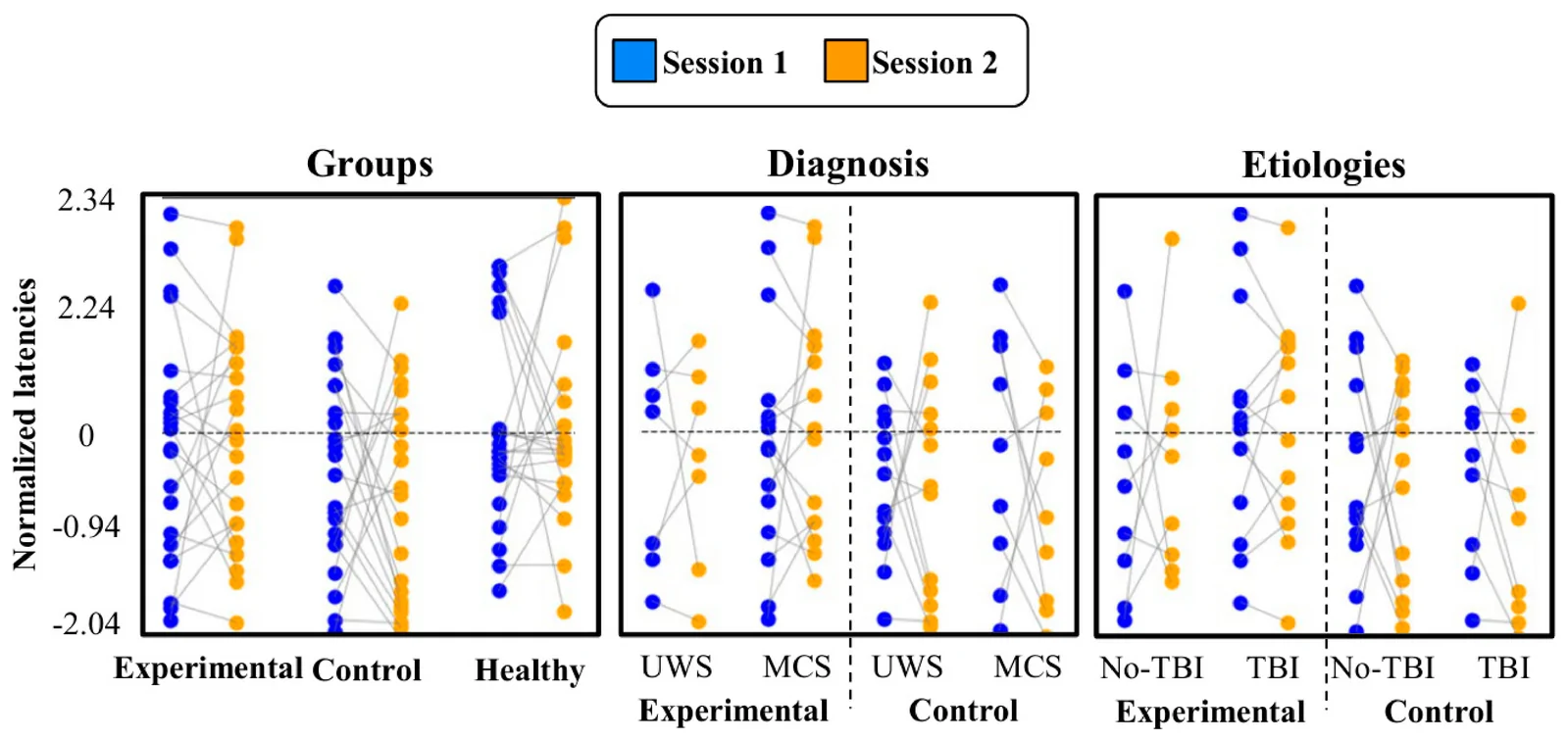
Distribution of MMN latencies across participants, categorized by group, diagnosis, and etiologies. Note: Values are z-score normalized relative to the healthy group. Data from both the first (blue) and second (orange) recording sessions are shown.

The average latency of the MMN component was 201 ± 75 ms following stimulus presentation, with no significant differences observed between the comparative groups, diagnoses, or etiologies, nor between the two evaluated sessions. Individual distributions normalized to the healthy group are available in Figure 10.

### 3.8. Safety Considerations

No adverse events associated with AVNT were observed in any of the included participants. However, one participant initially included in the experimental group experienced medical complications and an increase in seizure frequency before receiving the stimulation, while two participants in the control group experienced similar clinical events before completing the study. Another participant initially assigned to the experimental group died before completing all stimulation sessions due to causes unrelated to the treatment. All of these participants were withdrawn from the study at an early stage or before receiving the intervention and were therefore not included in the final sample. None of these events was considered attributable to AVNT.

## 4. Discussion

The primary aim of this study was to examine whether AVNT was associated with differential behavioral and neurophysiological trajectories in disorders of consciousness, as reflected by changes in total CRS-R scores and MMN responses across sessions, compared to neurotypical conditions. These patterns differed across diagnostic and etiological subgroups, highlighting the relevance of clinical heterogeneity in DoCs.

### 4.1. Demographic Characteristics and Timeframe

Demographic and temporal factors differed between groups and were considered as potential confounders.

The healthy group included a significantly higher proportion of women compared with the experimental and control groups, which may represent a potential confounding factor. Additionally, significant sex differences were observed in peak analyses across all study variables. In contrast, no significant sex-related differences were detected in peak-to-peak MMN measures after correction for multiple comparisons, suggesting that sex distribution was unlikely to fully explain the group differences observed in these analyses. Nevertheless, given the limited sample size and the unequal sex distribution across groups, the study may have been underpowered to detect small sex-related effects. It should be noted that previous studies have reported no sex-related differences in MMN amplitude or scalp topography, with sex effects being primarily associated with latency differences [49].

Likewise, the healthy group also showed a longer time interval between sessions than the experimental and control groups. Although good test–retest reliability of the MMN component over a one-year period has been reported in healthy individuals [50], this difference could be considered a potential confounding factor. After adjustment for the inter-session interval, overall group effects were observed for changes in peak-to-peak amplitude. Nevertheless, differences in session spacing remain a limitation when interpreting the longitudinal findings.

Finally, the longer time since injury in the experimental group compared with the control group should also be considered another important confounding factor. However, a longer time since injury has been associated with a poorer prognosis [51], which would not favor the experimental group. In this context, the observed outcomes would be compatible with a differential neurophysiological modulation associated with AVNT, rather than being solely explained by time since injury.

### 4.2. Classification by Groups

According to previous findings from our research group [23], no significant differences in total CRS-R scores were observed between the control and experimental groups during the first session. Both groups showed an increase in scores during the second session, which did not appear to be directly related to AVNT. Therefore, behavioral fluctuations and CRS-R improvements may also reflect the standard clinical care received by all patients, including routine multisensory stimulation, which is commonly implemented in DoC rehabilitation settings [52].

MMN results were interpreted relative to healthy reference distributions, using normalization by sex, ROI, and session [53]. Corresponding unnormalized analyses are presented in Supplementary Section S3.

In the first session, the absence of amplitude differences between groups may reflect preserved responsiveness in the clinical sample, similar to the neurotypical condition. Conversely, the control group showed a generalized decrease in MMN amplitude across sessions, which became significantly different from the neurotypical condition, potentially reflecting reduced efficiency in automatic auditory discrimination processes [33]. Although a decrease was also observed in the experimental group for the frontocentral region, it was not sufficient to produce a significant difference from the healthy group, suggesting the preservation of the underlying cognitive functions.

In addition, the analysis of between-session difference scores revealed significant differences between the control and healthy groups, whereas this pattern was not observed following AVNT exposure. These effects were observed in parieto-occipital regions. Although this area does not correspond to the canonical MMN topography, previous studies in cochlear implant users have suggested that the right temporo-parieto-occipital junction is involved in auditory spatial processing [54]. This pattern may reflect a gradual divergence of the control group from the neurotypical condition, potentially associated with difficulties in auditory spatial localization, whereas such divergence was not observed in the experimental group following AVNT exposure. However, these patterns do not appear to be sufficiently robust to distinguish between the two clinical groups, and therefore the evidence is insufficient to demonstrate a direct effect of AVNT stimulation.

Regarding the peak-to-peak approach, the results support the hypothesis that the MMN component is reduced in the absence of AVNT, a pattern not observed in the experimental group. Furthermore, this effect was more pronounced in the right hemisphere, which is associated with non-linguistic automatic auditory discrimination processes [55] and is widely implicated in the functional organization of the salience network [56]. In this context, the reduced MMN amplitude observed in the control group could be compatible with alterations in auditory deviance processing and related salience mechanisms. Moreover, given that the salience network has been reported to be impaired in DoCs and proposed as a potential target for AVNT-related restoration [9], the lateralized pattern observed in the present study and the lack of this effect in the experimental group could be compatible with a potential stimulation-related modulation.

Notably, the correlation between changes across sessions in CRS-R scores and MMN peak-to-peak amplitude observed in the experimental group could provide additional information regarding the relationship between neurophysiological modulation and behavioural changes. However, given that the correlations did not remain significant after accounting for multiple comparisons, coupled with the fact that the left region does not represent a canonical topography for MMN generation and that correlation does not imply causality, these results should be interpreted cautiously and cannot be considered definitive evidence of an AVNT effect.

### 4.3. Classification by Diagnoses and Etiologies

Given the limited sample size within each diagnostic and etiological group, these analyses should be interpreted as exploratory. However, they may provide information about how these conditions may influence AVNT effects.

The majority of effects in the MMN modulation were observed in the UWS diagnosis. Although stimulation effects have been more extensively reported in MCS [11], they may already present a relatively preserved MMN-related mechanism at baseline [57], which could limit the observable effect of AVNT. Regarding etiology, within-group MMN changes were observed in the control subgroup with TBI.

However, analyses of between-session change scores did not detect significant differences between groups within the diagnostic or etiological classifications. Therefore, these exploratory analyses did not demonstrate an AVNT-related difference in longitudinal trajectories.

### 4.4. Latency Analysis

No significant differences in MMN latency were observed across groups, diagnoses, or etiologies, suggesting that the temporal characteristics of automatic auditory processing [58] were comparable among the studied groups. These findings were consistent with previous reports indicating that MMN latency lacks prognostic or diagnostic value [57] and remains unchanged between coma and awakening states [59]. Similarly, studies evaluating MMN following AVNT in other pathologies, such as tinnitus, also reported no differences in latency [13]. Nevertheless, some studies described a shortening of MMN latency in patients with more favorable outcomes [60], highlighting the need for further research to clarify the clinical significance of MMN latency changes after AVNT stimulation.

### 4.5. Auricular Vagal Neuromodulation Therapy Protocols

There is currently no consensus on stimulation protocols for AVNT in DoCs, resulting in considerable heterogeneity in the parameters used and precluding the identification of an optimal approach [12].

For this reason, it is possible that alternative settings could have produced more pronounced effects on the CRS-R. In particular, a frequency of 25 Hz applied to the cymba for four hours daily over several months has been associated with greater improvements at the individual level [10]. However, these studies were based on very small sample sizes and lacked a control group without active neuromodulation, making it difficult to determine whether this approach was truly more effective.

In contrast, the protocol used in the current study, consisting of 20 Hz stimulation of the tragus delivered in two daily 30-min sessions over one month, has demonstrated effects in MCS at the group level in a larger sample size [11]. Additionally, these parameters were consistent with the ranges commonly reported in the literature [12].

### 4.6. Safety Considerations

No adverse events judged to be related to AVNT were observed in this study, which supports its preliminary safety in this cohort. However, due to the participants’ limited communication abilities and the absence of a standardized stimulation-specific tolerability measure, mild discomfort or a burning sensation at the stimulation site cannot be ruled out. Previous studies have described AVNT as generally safe and well tolerated, with predominantly mild and transient adverse effects. Furthermore, because AVNT is designed to activate afferent fibers of the auricular branch rather than directly stimulating cardiac vagal efferent fibers, the risk of clinically relevant cardiac effects is considered low [61,62].

### 4.7. Strengths

The study integrates CRS-R assessments with analyses of MMN latency, peak amplitude, and peak-to-peak amplitude, including exploratory comparisons by diagnosis and etiology, in line with multimodal assessment recommendations [21,22]. It builds on the prognostic relevance of MMN [57] and previous AVNT research using neurophysiological biomarkers [63]. Normalization to the healthy group provides a neurotypical reference [53], and detailed reporting of the protocol supports replication.

### 4.8. Limitations

First, the characteristics of the clinical population posed several challenges in controlling methodological factors, including difficulties with blinding and limitations in randomizing participants into control or experimental groups. The non-randomized formation of the experimental and control groups may have introduced selection bias and limited causal interpretation of the findings. Additionally, in the absence of a sham-stimulation condition, nonspecific procedural effects, such as transient changes in arousal associated with device placement and handling, cannot be ruled out. These circumstances were largely due to the study being conducted in a healthcare center where all patients required active treatment, making strict experimental control unfeasible. Similarly, it was not possible to conduct a proper follow-up of the patients, leaving the duration of the effects of AVNT unknown. In addition, participants in the clinical groups received routine therapeutic activities and pharmacotherapy, which were not part of the experimental design, so the specific effect of AVNT could not be isolated. However, these routine activities were consistent across participants, providing a comparable clinical context between groups, although the specific effects of AVNT should be interpreted in the context of concurrent ongoing interventions.

Furthermore, the experimental and control groups differed in the CRS-R stability criterion applied before assessment. This approach was adopted to minimize the influence of spontaneous clinical fluctuations in the experimental group. However, this methodological difference may limit direct comparability between groups and may have contributed to the observed differences in MMN trajectories. Therefore, this aspect should be taken into consideration when interpreting the findings, although no significant differences were observed between groups in CRS-R scores at baseline [23].

According to the required sample size, it was calculated a priori for a mixed ANOVA, although the statistical power may be affected by the use of non-parametric tests due to violations of normality and homoscedasticity. Similarly, under these conditions, the non-parametric tests did not allow for a straightforward assessment of the interaction effects between session and group, which may be important for a better understanding of the influence of AVNT. However, this limitation was addressed by incorporating between-session difference scores into the analysis.

Moreover, even when an adequate sample size was achieved at the group level, further stratification by diagnosis or etiology resulted in small comparison groups due to the challenges associated with recruiting participants with DoCs. This limitation may have affected the findings by reducing statistical power and increasing the risk of underestimating potential effects. Nevertheless, given the considerable heterogeneity of diagnostic and etiological profiles within this cohort, accounting for these factors remains essential, as it may provide valuable insights into their influence on the effectiveness of AVNT.

Regarding the electroencephalographic recording, the use of an eight-electrode cap introduced certain inherent technical limitations. Consequently, the limited number of electrodes constrained the characterization of MMN topography and reduced the spatial resolution of the recordings, thereby limiting the interpretation of fine-grained regional differences. Nevertheless, given the clinical characteristics of the sample, a simple and rapid cap montage was selected as a pragmatic compromise given the clinical context. Similarly, skull defects are common in patients with severe brain injury and could distort electroencephalographic recordings by altering impedances and signal propagation, thereby impacting signal quality and interpretation. Furthermore, although MMN peak detection was standardized according to its typical characteristics and visually verified, extending the canonical time window to later latencies could increase the risk of capturing other unrelated event-related potential components.

Although normalization based on the healthy group allowed clinical values to be expressed relative to the corresponding neurotypical reference distribution, converting the data to z-scores entails certain methodological limitations. Because the healthy group served as the reference for the normalization procedure, its inclusion in subsequent between-group comparisons may have introduced a degree of statistical dependence that should be considered when interpreting these findings. Nevertheless, retaining the healthy group in these analyses allowed for the determination of whether the clinical groups significantly deviated from the neurotypical reference at the group level. Importantly, the consistency between the normalized analyses and the corresponding unnormalized analyses presented in Supplementary Section S3 suggested that the main findings were robust to the normalization procedure. Although normalization facilitated interpretation by expressing results relative to the neurotypical distribution and enabled a more precise characterization of group-specific effects, the overall pattern of results was partially consistent with the unnormalized analyses. This partial convergence may suggest that any statistical dependence arising from the normalization procedure did not meaningfully influence the observed group differences.

The longer interval between assessment sessions in the healthy group compared with the clinical groups may also have affected the interpretation of the longitudinal findings. However, the good test–retest reliability of MMN amplitude over a one-year period in healthy individuals [50], together with the absence of significant between-session differences in the healthy group, suggests that the impact of this discrepancy was likely limited. Overall, these methodological considerations should be taken into account when interpreting the findings.

Finally, although the available medical records, clinical reports, and anamnesis did not indicate clinically relevant hearing impairment, hearing function was not formally assessed through audiological or electrophysiological testing. Therefore, adequate auditory function could not be confirmed. Given that the primary outcome relies on auditory stimulus processing, this limitation should be considered when interpreting the findings. In addition, because the presentation intensity (dB SPL) was not measured and no sound level calibration was performed due to clinical constraints, it could not be ensured that all participants perceived the auditory stimuli at the same sound intensity. Although the distance between the speakers and the participant was maintained at 70–80 cm, this alone did not ensure consistent sound intensity across participants.

Despite these limitations, the present study provides ecologically valid neurophysiological evidence obtained in a real-world clinical setting, offering insights into AVNT-associated trajectories that would be difficult to capture under strictly controlled experimental conditions.

### 4.9. Future Research Directions

The present study could provide a basis for future neurophysiological investigations into consciousness recovery in DoCs, particularly by focusing on automatic auditory discrimination following AVNT.

Future studies should also explicitly consider the stage of disorders of consciousness (subacute versus prolonged/chronic), as the effects of neuromodulatory interventions may vary depending on the temporal distance from injury. Moreover, the imbalance in clinical stability between the study groups could be minimized by implementing randomized or stratified allocation procedures and using mixed-effects models that include clinical stability as a covariate. Similarly, future studies should include a sham-stimulation condition involving identical procedures without active current delivery to control for nonspecific procedural effects and strengthen internal validity.

At the same time, high-density EEG combined with source localization methods such as sLORETA would allow a more accurate characterization of MMN topography and the regional effects of AVNT, particularly in patients with DoCs, where atypical spatial distributions may occur.

Furthermore, although this research focused on MMN, other event-related potential components would also be important for studying possible attentional and cognitive improvements after AVNT, such as P3a, P3b, N400, or P600 [16]. These components would enable the investigation of higher-order attentional and cognitive processes beyond early sensory mismatch detection. Similarly, resting-state spectral analyses [64] and event-related potential wavelet analyses [65], together with connectivity [66] and entropy [67] measures, could provide deeper insights into the effects of AVNT on large-scale brain networks and consciousness recovery. However, these approaches typically require higher spatial resolution, longer recordings, or dedicated analytical pipelines, which were not available in the present study.

Other peripheral physiological measures, such as heart rate variability, pupillometry, and galvanic skin conductance, could be incorporated in future studies to provide a multimodal assessment of autonomic function. This would help validate the proposed vagal mechanism of AVNT and determine whether its effects are mediated by activation of the autonomic nervous system [68].

In addition, other statistical approaches could be especially useful for future studies with larger samples or longer longitudinal follow-up, such as linear mixed models, bootstrap methods, or Bayesian models.

Finally, this study highlights the growing need to improve diagnostic and prognostic methods in DoCs by integrating etiological and neurophysiological information within a multimodal framework [21,22].

## 5. Conclusions

The current research provides new insights into how AVNT may modulate the MMN component as a marker of automatic auditory processing in DoCs. Although both the experimental and control groups exhibited higher total CRS-R scores during the second session, MMN analysis revealed distinct patterns of change between the groups relative to the neurotypical condition. Participants in the non-stimulated control group exhibited distinct trajectories in MMN amplitude compared with the neurotypical group, potentially reflecting impaired automatic auditory discrimination and spatial processing. In contrast, this pattern was not observed in participants who underwent forty AVNT sessions. Although this finding is compatible with a possible effect of AVNT, the evidence remains insufficient to demonstrate a direct effect of the intervention. Further studies are required to address the intrinsic limitations associated with this clinical population and to determine whether AVNT produces clinically meaningful changes in auditory processing.

## Figures and Tables

**Table 1:** Potential confounding variables affecting the association between AVNT and electroencephalographic measures.

	Experimental	Control	Healthy
Size	22	22	22
Sex	17 men/5 women	14 men/8 women	7 men/15 women (#)
Diagnostic distribution	15 MCS/7 UWS	9 MCS/13 UWS	-
Etiology distribution	13 TBI/9 No-TBI	13 TBI/9 No-TBI	-
Age	40 ± 17	34 ± 17	33 ± 10
Initial CRS-R	10 ± 4	8 ± 3	-
Months since injury	9 ± 4 (#)	6 ± 4 (#)	-
Days between sessions	53 ± 22 (#)	68 ± 51	121 ± 80 (#)

Note: The hashtag symbol (#) represents significant differences between the two groups, while a symbol appearing only in one group indicates that this group is significantly different from the others.

**Table 2:** CRS-R scores by groups and sessions.

	Session 1	Session 2	Change
Experimental	10 ± 4 (*)	11 ± 5 (*)	1 ± 2
Control	8 ± 3 (*)	10 ± 5 (*)	2 ± 2

Note: The asterisk symbol (*) represents significant differences between sessions (Wilcoxon test).

**Table 3:** Normalized amplitude peak values of the MMN component according to the healthy group.

	Session	Fronto-Central ($\bar{x}$ ± SD)	Parieto-Occipital ($\bar{x}$ ± SD)	Left ($\bar{x}$ ± SD)	Right ($\bar{x}$ ± SD)
Experimental	First session	−0.77 ± 2.40 (*)(†)	−0.38 ± 1.90	−0.34 ± 2.48	−0.36 ± 3.38 (†)
	Second session	0.67 ± 1.34 (*)	0.56 ± 1.04	0.79 ± 1.08	0.67 ± 1.60
	Difference	1.44 ± 2.17	0.94 ± 2.07	1.13 ± 2.73	1.03 ± 3.62
Control	First session	−0.23 ± 1.62 (*)	−0.45 ± 2.08 (*)	−0.26 ± 1.86 (*)	−0.28 ± 1.88 (*)
	Second session	1.00 ± 1.26 (*)(#)	1.02 ± 1.14 (*)(#)	1.18 ± 1.10 (*)(#)	1.24 ± 1.13 (*)(#)
	Difference	1.23 ± 2.13	1.47 ± 2.67 (#)	1.44 ± 2.51	1.52 ± 2.43
Healthy	First session	0.00 ± 0.84	0.00 ± 0.92	0.00 ± 0.87	0.0 ± 0.88
	Second session	0.00 ± 0.76 (#)	0.00 ± 0.74 (#)	0.00 ± 0.76 (#)	0.00 ± 0.73 (#)
	Difference	0.00 ± 1.44	0.00 ± 1.45 (#)	0.00 ± 1.37	0.00 ± 1.44

Note: The asterisk symbol (*) represents significant differences between sessions (Wilcoxon test), whereas the hashtag symbol (#) indicates significant differences between groups (Kruskal–Wallis and Mann–Whitney U tests) and the dagger symbol (†) marks differences between regions (Friedman and Wilcoxon tests). The significance level for all tests was set at *p*_Bonferroni_ < 0.05.

**Table 4:** Normalized peak-to-peak amplitude values of the MMN component according to the healthy group.

	Session	Fronto-Central ($\bar{x}$ ± SD)	Parieto-Occipital ($\bar{x}$ ± SD)	Left ($\bar{x}$ ± SD)	Right ($\bar{x}$ ± SD)
Experimental	First session	−0.33 ± 0.68	0.62 ± 2.12	0.26 ± 1.80	−0.15 ± 1.02
	Second session	−0.06 ± 0.84	0.15 ± 1.48	0.16 ± 1.68	0.12 ± 1.88
	Difference	0.27 ± 1.22	−0.47 ± 2.50	−0.10 ± 2.59	0.27 ± 1.87
Control	First session	−0.20 ± 0.84	1.19 ± 2.53 (*)(†)	0.25 ± 1.34 (*)	0.15 ± 1.03 (*)
	Second session	−0.25 ± 1.04	−0.44 ± 0.97 (*)	−0.47 ± 0.88 (*)	−0.74 ± 0.72 (*)(#)
	Difference	−0.05 ± 1.11	−1.63 ± 2.90 (#)	−0.72 ± 1.17	−0.89 ± 1.31
Healthy	First session	0.00 ± 0.73	0.00 ± 0.67	0.00 ± 0.79	0.00 ± 0.75
	Second session	0.00 ± 0.96	0.00 ± 0.78	0.00 ± 0.84	0.00 ± 0.87 (#)
	Difference	0.00 ± 1.32	0.00 ± 1.19 (#)	0.00 ± 1.05	0.00 ± 1.24

Note: The asterisk symbol (*) represents significant differences between sessions (Wilcoxon test), whereas the hashtag symbol (#) indicates significant differences between groups (Kruskal–Wallis and Mann–Whitney U tests) and the dagger symbol (†) marks differences between regions (Friedman and Wilcoxon tests). The significance level for all tests was set at *p*_Bonferroni_ < 0.05.

**Table 5:** CRS-R scores by diagnosis and sessions.

	Session 1	Session 2	Change
Experimental with UWS	6 ± 1	6 ± 1	1 ± 1
Experimental with MCS	12 ± 3 (*)	14 ± 4 (*)	2 ± 2
Control with UWS	6 ± 1	7 ± 1	1 ± 1
Control with MCS	11 ± 3 (*)	14 ± 5 (*)	3 ± 3

Note: The asterisk symbol (*) represents significant differences between sessions (Wilcoxon test).

**Table 6:** Normalized amplitude peak values of the MMN component according to the healthy group, by diagnosis.

	Session	Fronto-Central ($\bar{x}$ ± SD)	Parieto-Occipital ($\bar{x}$ ± SD)	Left ($\bar{x}$ ± SD)	Right ($\bar{x}$ ± SD)
Experimental with UWS	First session	−0.36 ± 2.42	−0.27 ± 1.92	−0.16 ± 2.44	0.36 ± 2.64
	Second session	0.66 ± 1.69	0.88 ± 1.47	1.38 ± 1.30	0.89 ± 2.23
	Difference	1.02 ± 2.80	1.15 ± 2.46	1.54 ± 3.15	0.53 ± 3.51
Experimental with MCS	First session	−0.96 ± 2.46 (*)	−0.42 ± 1.96	−0.43 ± 2.57	−0.69 ± 3.72
	Second session	0.67 ± 1.21 (*)	0.41 ± 0.78	0.52 ± 0.89	0.57 ± 1.28
	Difference	1.63 ± 2.83	0.83 ± 1.96	0.95 ± 2.61	1.26 ± 3.76
Control with UWS	First session	−0.43 ± 1.43 (*)	−0.67 ± 2.11 (*)	−0.42 ± 1.86 (*)	−0.53 ± 1.71 (*)
	Second session	1.39 ± 1.30 (*)	1.13 ± 1.13 (*)	1.36 ± 1.03 (*)	1.54 ± 1.03 (*)
	Difference	1.82 ± 2.15	1.80 ± 2.90	1.78 ± 2.73	2.07 ± 2.42
Control with MCS	First session	0.05 ± 1.92	−0.13 ± 2.12	−0.02 ± 1.95	0.10 ± 2.16
	Second session	0.44 ± 1.00 (†)	0.87 ± 1.21	0.92 ± 1.21	0.80 ± 1.17 (†)
	Difference	0.39 ± 1.92	1.00 ± 2.38	0.94 ± 2.20	0.70 ± 2.33

Note: The asterisk symbol (*) represents significant differences between sessions (Wilcoxon test), and the dagger symbol (†) marks differences between regions (Friedman and Wilcoxon tests). The significance level for all tests was set at *p*_Bonferroni_ < 0.05.

**Table 7:** Normalized peak-to-peak amplitude values of the MMN component according to the healthy group, by diagnoses.

	Session	Fronto-Central ($\bar{x}$ ± SD)	Parieto-Occipital ($\bar{x}$ ± SD)	Left ($\bar{x}$ ± SD)	Right ($\bar{x}$ ± SD)
Experimental with UWS	First session	−0.39 ± 0.70	0.78 ± 3.08	0.39 ± 2.56	−0.36 ± 0.96
	Second session	−0.46 ± 0.45	−0.29 ± 1.24	−0.73 ± 0.66	−0.40 ± 1.11
	Difference	−0.07 ± 1.12	−1.07 ± 3.00	−1.12 ± 2.29	−0.04 ± 1.61
Experimental with MCS	First session	−0.30 ± 0.70	0.54 ± 1.62	0.20 ± 1.42	−0.06 ± 1.06
	Second session	0.12 ± 0.93	0.35 ± 1.57	0.58 ± 1.86	0.36 ± 2.14
	Difference	0.42 ± 1.27	−0.19 ± 2.30	0.38 ± 2.65	0.42 ± 2.02
Control with UWS	First session	−0.18 ± 0.84	1.74 ± 3.14 (*)(†)	0.44 ± 1.25 (*)	0.19 ± 1.13
	Second session	−0.31 ± 0.90	−0.38 ± 0.96 (*)	−0.50 ± 0.63 (*)	−0.68 ± 0.68
	Difference	−0.13 ± 1.01	−2.12 ± 3.58	−0.94 ± 1.34	−0.87 ± 1.41
Control with MCS	First session	−0.24 ± 0.89	0.40 ± 0.86	−0.03 ± 1.49	0.09 ± 0.92
	Second session	−0.18 ± 1.27	−0.52 ± 1.04	−0.42 ± 1.19	−0.83 ± 0.80
	Difference	0.06 ± 1.31	−0.92 ± 1.41	−0.39 ± 0.85	−0.92 ± 1.25

Note: The asterisk symbol (*) represents significant differences between sessions (Wilcoxon test), and the dagger symbol (†) marks differences between regions (Friedman and Wilcoxon tests). The significance level for all tests was set at *p*_Bonferroni_ < 0.05.

**Table 8:** CRS-R scores by etiology and sessions.

	Session 1	Session 2	Change
Experimental with No-TBI	8 ± 4	9 ± 4	1 ± 1
Experimental with TBI	11 ± 4 (*)	13 ± 5 (*)	2 ± 2
Control with No-TBI	6 ± 1	8 ± 3	1 ± 2
Control with TBI	9 ± 4 (*)	11 ± 6 (*)	2 ± 2

Note: The asterisk symbol (*) represents significant differences between sessions (Wilcoxon test).

**Table 9:** Normalized amplitude peak values of the MMN component according to the healthy group, by etiologies.

	Session	Fronto-Central ($\bar{x}$ ± SD)	Parieto-Occipital ($\bar{x}$ ± SD)	Left ($\bar{x}$ ± SD)	Right ($\bar{x}$ ± SD)
Experimental with No-TBI	First session	0.00 ± 2.31	−0.06 ± 2.01	0.07 ± 2.51	0.33 ± 2.39
	Second session	0.79 ± 1.23 (†)	0.84 ± 1.03	1.26 ± 1.11 (†)	0.88 ± 1.54
	Difference	0.79 ± 2.64	0.90 ± 2.23	1.19 ± 3.10	0.55 ± 2.89
Experimental with TBI	First session	−0.83 ± 1.80 (†)	−0.18 ± 1.18	−0.12 ± 1.79	0.20 ± 1.47 (†)
	Second session	0.59 ± 1.52	0.47 ± 1.02	0.53 ± 0.99	0.59 ± 1.74
	Difference	1.42 ± 2.86	0.65 ± 2.06	0.65 ± 2.59	0.39 ± 2.34
Control with No-TBI	First session	0.35 ± 1.30	−0.71 ± 2.48	0.12 ± 1.74	−0.47 ± 1.90
	Second session	0.91 ± 0.92	1.35 ± 1.42	1.28 ± 1.26	1.39 ± 1.20
	Difference	0.56 ± 1.62	2.06 ± 3.61	1.16 ± 2.79	1.86 ± 2.80
Control with TBI	First session	−0.64 ± 1.74 (*)	−0.27 ± 1.85	−0.52 ± 1.97 (*)	−0.14 ± 1.94
	Second session	1.06 ± 1.48 (*)	0.80 ± 0.90	1.11 ± 1.02 (*)	1.13 ± 1.11
	Difference	1.70 ± 2.38	1.07 ± 1.83	1.63 ± 2.39	1.27 ± 2.39

Note: The asterisk symbol (*) represents significant differences between sessions (Wilcoxon test), and the dagger symbol (†) marks differences between regions (Friedman and Wilcoxon tests). The significance level for all tests was set at *p*_Bonferroni_ < 0.05.

**Table 10:** Normalized peak-to-peak amplitude values of the MMN component according to the healthy group, by etiologies.

	Session	Fronto-Central ($\bar{x}$ ± SD)	Parieto-Occipital ($\bar{x}$ ± SD)	Left ($\bar{x}$ ± SD)	Right ($\bar{x}$ ± SD)
Experimental with No-TBI	First session	−0.50 ± 0.68	0.47 ± 2.64	0.22 ± 2.26	−0.33 ± 0.76
	Second session	−0.39 ± 0.87	−0.39 ± 1.47	−0.57 ± 1.16	−0.64 ± 1.40
	Difference	0.11 ± 1.28	−0.86 ± 2.87	−0.79 ± 2.57	−0.31 ± 1.63
Experimental with TBI	First session	−0.22 ± 0.69	0.72 ± 1.78	0.29 ± 1.50	−0.03 ± 1.18
	Second session	0.16 ± 0.77	0.52 ± 1.42	0.67 ± 1.83	0.65 ± 2.04 (#)
	Difference	0.38 ± 1.21	−0.20 ± 2.30	0.38 ± 2.59	0.68 ± 1.99
Control with No-TBI	First session	−0.28 ± 0.84	2.00 ± 3.70 (†)	0.33 ± 1.28	0.16 ± 1.22
	Second session	−0.17 ± 0.99	−0.58 ± 0.80	−0.46 ± 0.55	−0.59 ± 0.71
	Difference	0.11 ± 1.16	−2.58 ± 4.14	−0.79 ± 1.42	−0.75 ± 1.52
Control with TBI	First session	−0.15 ± 0.88	0.63 ± 1.10 (*)	0.19 ± 1.43 (*)	0.15 ± 0.93 (*)
	Second session	−0.31 ± 1.11	−0.34 ± 1.09 (*)	−0.48 ± 1.07 (*)	−0.84 ± 0.74 (*)(#)
	Difference	−0.16 ± 1.11	−0.97 ± 1.48	−0.67 ± 1.03	−0.99 ± 1.21

Note: The asterisk symbol (*) represents significant differences between sessions (Wilcoxon test), whereas the hashtag symbol (#) indicates significant differences between groups (Kruskal–Wallis and Mann–Whitney U tests) and the dagger symbol (†) marks differences between regions (Friedman and Wilcoxon tests). The significance level for all tests was set at *p*_Bonferroni_ < 0.05.

## Data Availability

The data supporting the findings of this study are not publicly available due to ethical and privacy restrictions involving a vulnerable clinical population. De-identified data may be made available from the corresponding author upon reasonable request and subject to approval by the relevant ethics committees and data-sharing agreements.

## Supplementary Materials

The following supporting information can be downloaded at: https://www.mdpi.com/article/10.3390/brainsci16101072/s1, Figure S1: Temporal structure of the study across the three groups; Figure S2: Electroencephalographic data processing protocol for the passive auditory oddball paradigm; Figure S3: Flowchart of the electroencephalographic statistical analysis pipeline; Figure S4: Difference waveforms for mismatch negativity during the passive auditory oddball paradigm; Figure S5: Event-related potential waveforms elicited during the passive auditory oddball paradigm for standard and deviant stimuli; Table S1: Demographic and clinical characteristics of participants in the experimental, control, and healthy groups; Table S2: Timeframe of the study; Table S3: Mean retained epochs after artifact rejection for each group and condition; Table S4: Participants identified as outliers based on the classification and type of analysis performed, along with the sample size for each comparative group; Table S5: Mean peak amplitude of the MMN component; Table S6: Mean peak-to-peak amplitude of the MMN component; Table S7: Mean peak amplitude of the MMN component, by diagnosis; Table S8: Mean peak-to-peak amplitude of the MMN component, by diagnosis; Table S9: Mean peak amplitude of the MMN component, by etiologies; Table S10: Mean peak-to-peak amplitude of the MMN component, by etiologies; Supplementary Sections S3 and S4: additional electrophysiological results and demographic analyses.

## GenAI Declaration

During the preparation of this manuscript, the authors used ChatGPT GPT-5.6 Luna, Copilot Version 2026.6.3, Consensus 2.0, NotebookLM on Gemini 3.5 for the purposes of addressing grammar issues, correcting statistical scripts, searching for scientific references, and organizing ideas, always with the authors’ own judgment. The authors have reviewed and edited the output and take full responsibility for the content of this publication.

## Abbreviations

The following abbreviations are used in this manuscript:

AVNT	Auricular Vagal Neuromodulation Therapy
CRS-R	Coma Recovery Scale Revised
DoCs	Disorders of Consciousness
ICA	Independent component analysis
IIR	Infinite impulse response
MAD	Median Absolute Deviation
MCS	Minimally Conscious State
MMN	Mismatch Negativity
No-TBI	Non-Traumatic Brain Injury
ROIs	Regions of interest
rTMS	Repetitive transcranial magnetic stimulation
TBI	Traumatic Brain Injury
tDCS	Transcranial direct current stimulation
UWS	Unresponsive Wakefulness Syndrome
