# Supplementary material for: Maintenance of Auditory Deviance Processing Following Auricular Vagal Neuromodulation Therapy in Disorders of Consciousness
Source: Brain Sci. 2026 Oct 6;16(10):1072. doi: 10.3390/brainsci16101072 (PMC13702590; doi:10.3390/brainsci16101072)
Supplement: Supplementary file 1 [file brainsci-16-01072-s001.zip › brainsci-4473844-supplementary.pdf]

Section S1 (Supplementary figures)

Figure S1. Temporal structure of the study across the three groups.

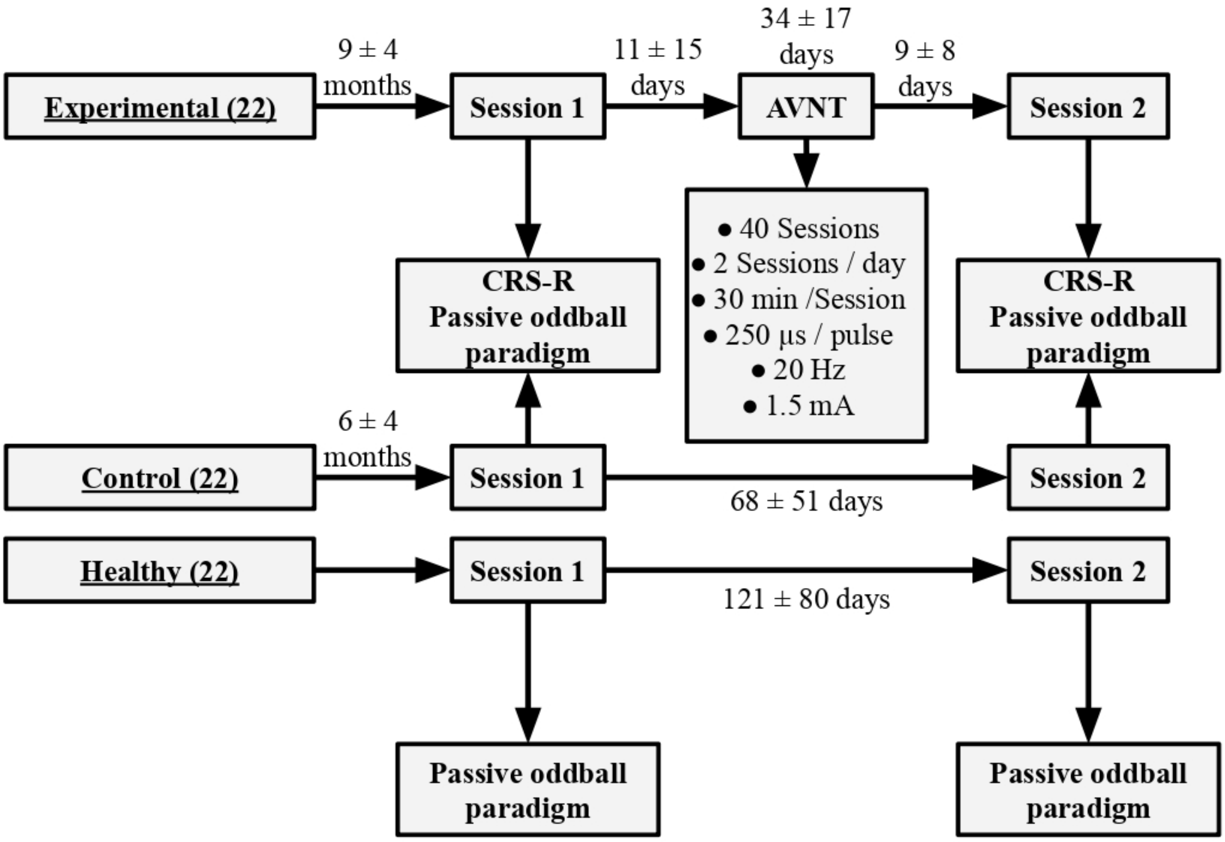

Figure S2. Electroencephalographic data processing protocol for the passive auditory oddball paradigm.

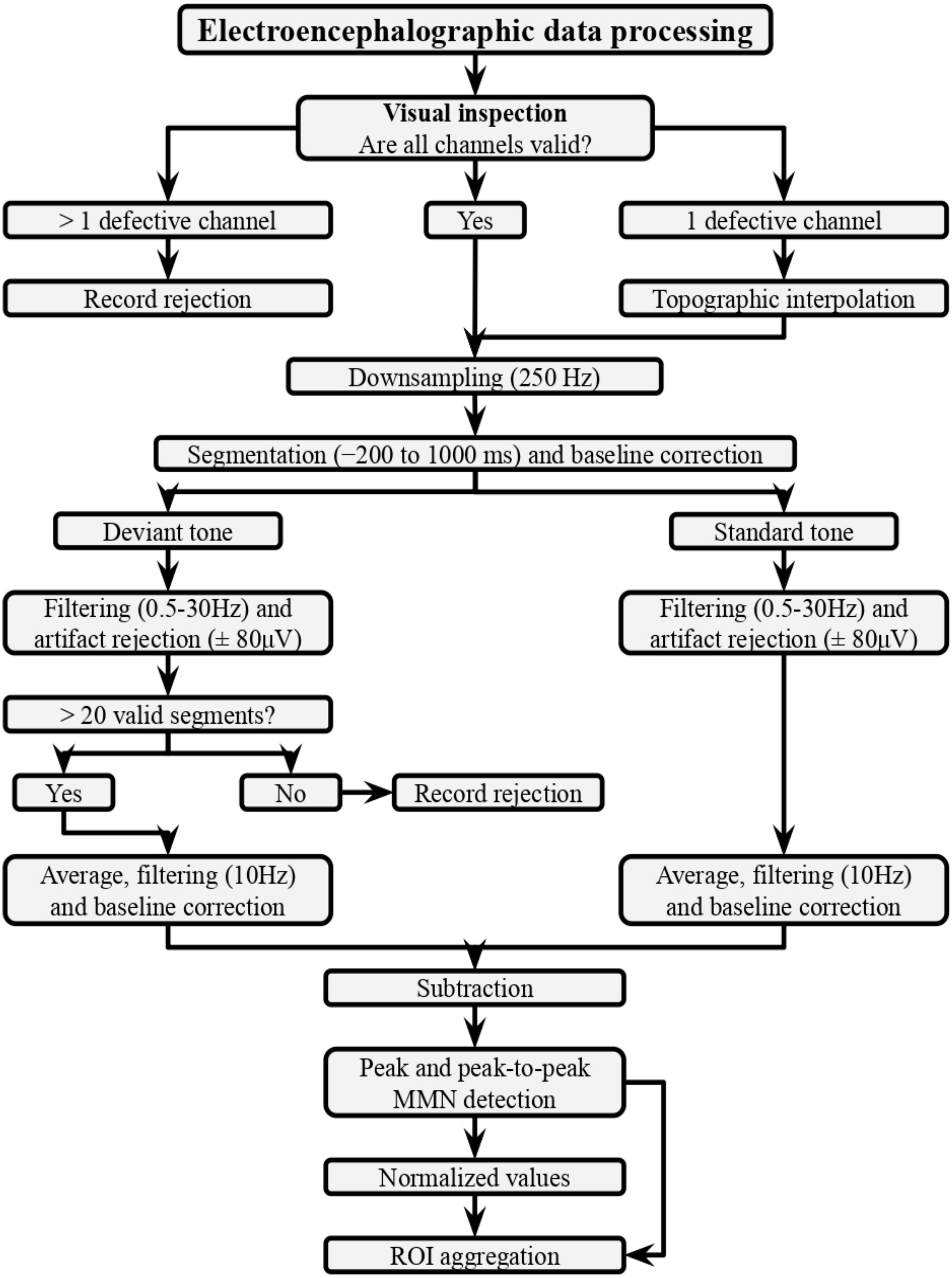

Figure S3. Flowchart of the electroencephalographic statistical analysis pipeline.

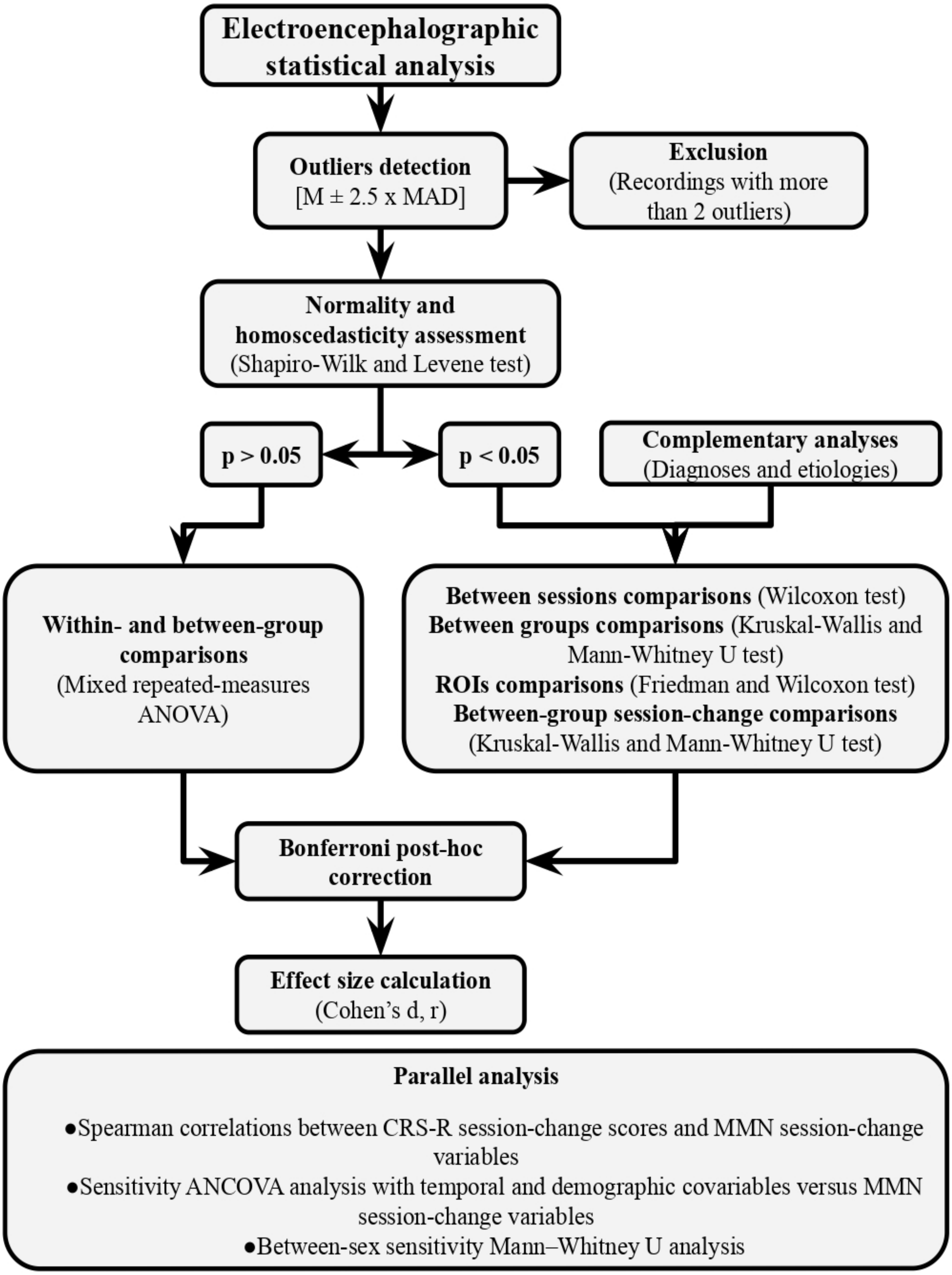

Figure S4. Difference waveforms for mismatch negativity during the passive auditory oddball paradigm.

Note: The waveform was computed as the difference wave obtained by subtracting the event-related potentials elicited by standard stimuli from those elicited by deviant stimuli, following the conventional procedure for mismatch negativity analysis.

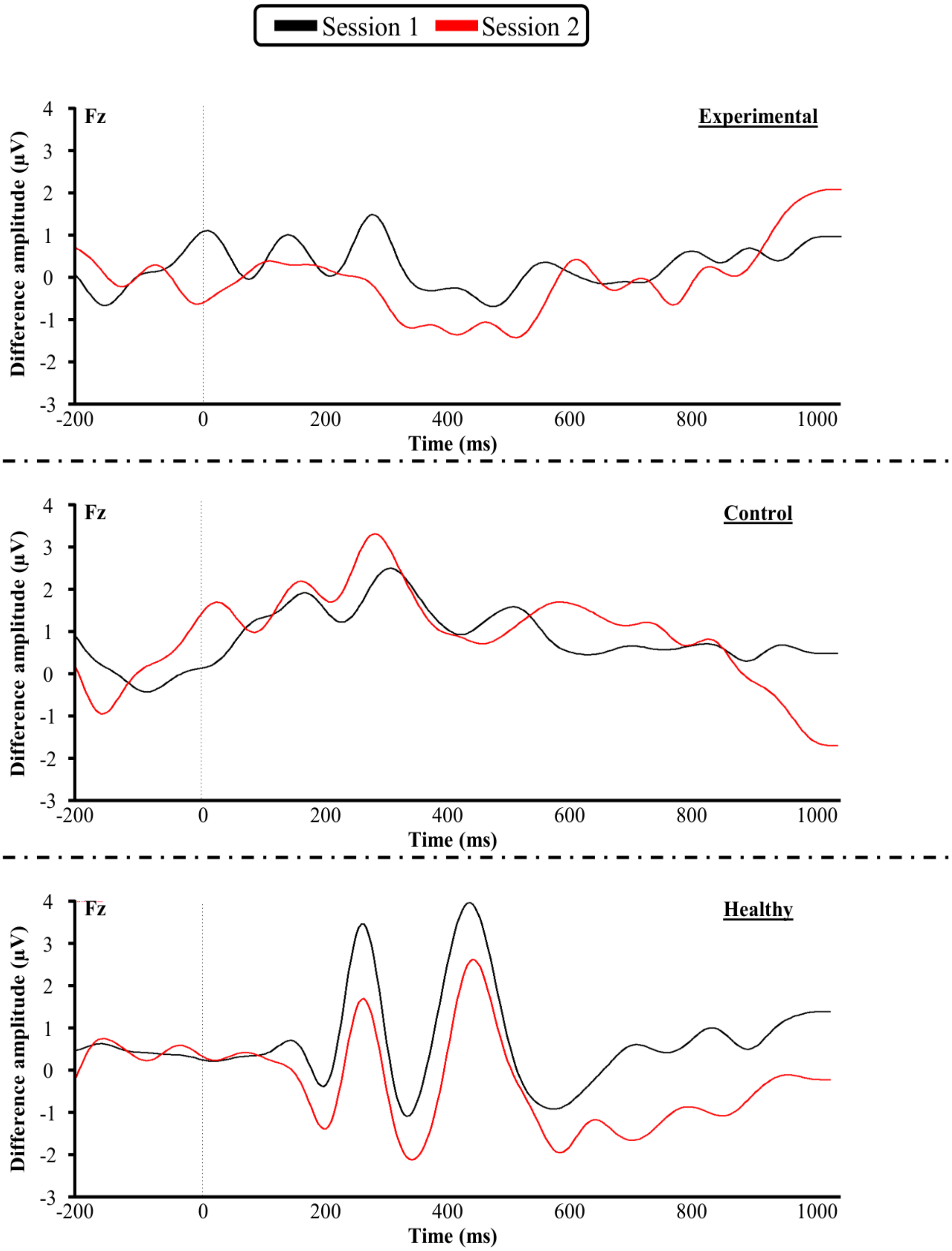

Figure S5. Event-related potential waveforms elicited during the passive auditory oddball paradigm for standard and deviant stimuli.

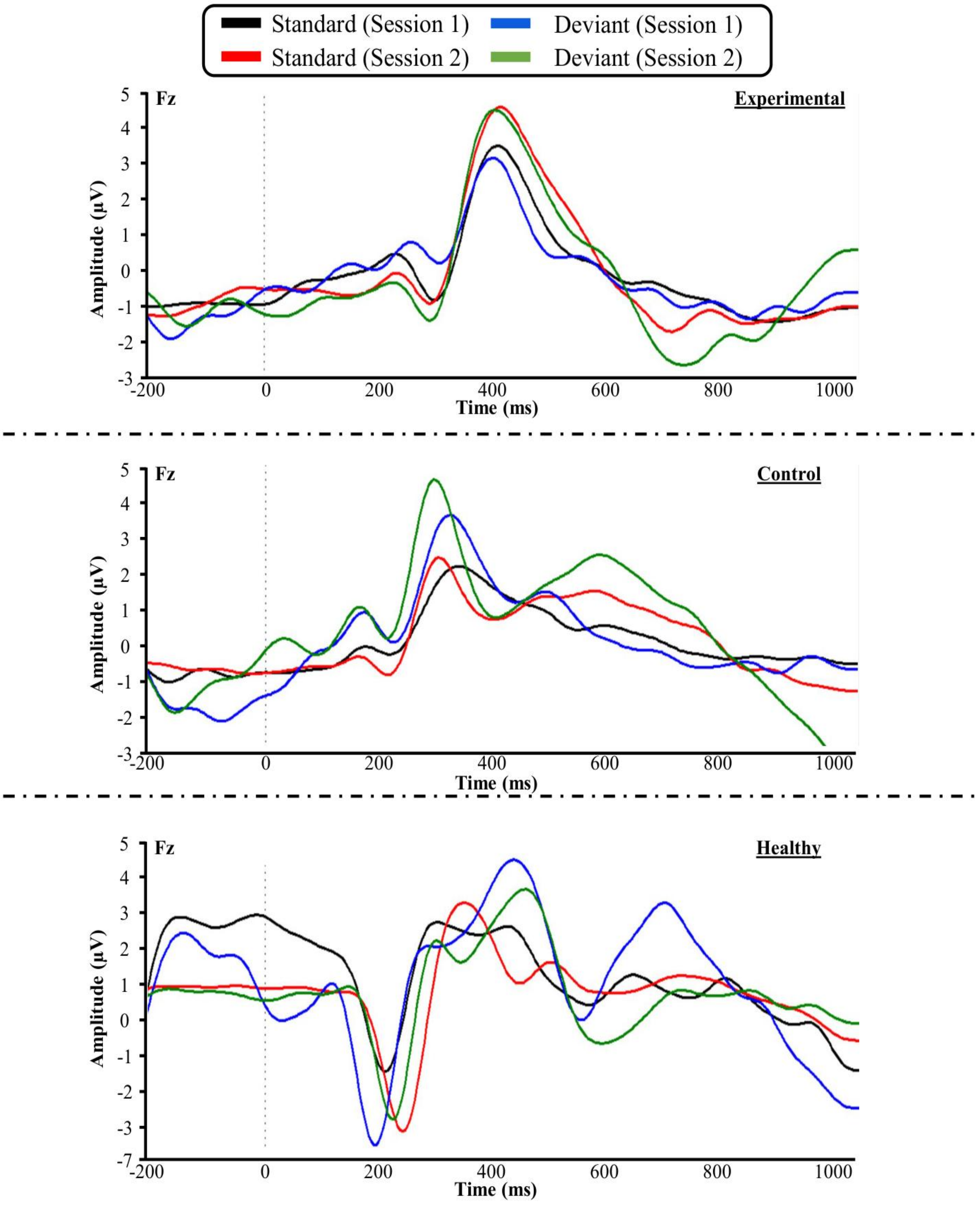

Section S2 (Supplementary tables)

Table S1. Demographic and clinical characteristics of participants in the experimental, control, and healthy groups.

Nº	Etiology	Diagnosis	Drugs consumption	Skull defects	Age	Sex	Group
**1**	No-TBI	UWS	Levetiracetam, Baclofen, Trazodone, Lorazepam	No	55	Men	Experimental
**2**	TBI	MCS	Trazodone, Levetiracetam, Lacosamide, Pregabalin, Aripiprazole	Yes	38	Men	Experimental
**3**	TBI	MCS	Baclofen, Levetiracetam, Escitalopram, Pregabalin	Yes	47	Men	Experimental
**4**	TBI	MCS	Levetiracetam, Clonazepam, Trazodone, Amantadine	Yes	32	Men	Experimental
**5**	No-TBI	UWS	Trazodone, Baclofen, Levetiracetam, Clonazepam	No	66	Men	Experimental
**6**	No-TBI	UWS	Levetiracetam, Baclofen, Gabapentin	No	50	Men	Experimental
**7**	TBI	MCS	Escitalopram, Baclofen, Tizanidine	Yes	46	Women	Experimental
**8**	No-TBI	MCS	Baclofen	No	59	Women	Experimental
**9**	No-TBI	MCS	Escitalopram, Clonazepam, Topiramate, Baclofen	No	69	Women	Experimental
**10**	No-TBI	UWS	Levetiracetam, Baclofen, Trazodone, Lorazepam	No	45	Men	Experimental
**11**	No-TBI	MCS	Levetiracetam, Baclofen, Amantadine	Yes	43	Men	Experimental
**12**	TBI	UWS	Levetiracetam, Trazodone, Clonazepam, Baclofen, Gabapentin, Levodopa	Yes	20	Men	Experimental
**13**	TBI	MCS	Levetiracetam, Amantadine, Escitalopram, Lacosamide	Yes	44	Men	Experimental
**14**	TBI	MCS	Levetiracetam, Baclofen, Clonazepam	Yes	24	Men	Experimental
**15**	TBI	MCS	Levetiracetam, Baclofen, Trazodone, Tizanidine	No	35	Men	Experimental
**16**	No-TBI	MCS	Baclofen, Levetiracetam	No	40	Men	Experimental
**17**	TBI	UWS	Baclofen, Clonazepam, Levetiracetam	No	12	Men	Experimental
**18**	TBI	UWS	Tizanidine, Levetiracetam, Amantadine, Clonazepam, Baclofen	Yes	25	Men	Experimental
**19**	TBI	MCS	Levetiracetam, Amantadine, Clonazepam, Escitalopram	Yes	72	Women	Experimental
**20**	TBI	MCS	Trazodone, Levetiracetam, Baclofen, Clonazepam	Yes	21	Men	Experimental
**21**	TBI	MCS	Trazodone, Baclofen, Clonazepam	No	14	Women	Experimental
**22**	No-TBI	MCS	Levetiracetam, Clonazepam	No	25	Men	Experimental
**23**	TBI	UWS	Levetiracetam, Lacosamide, Levodopa, Baclofen, Clonazepam	No	19	Men	Control
**24**	No-TBI	UWS	Brivaracetam, Valproate, Baclofen	No	37	Women	Control
**25**	No-TBI	UWS	Baclofen, Tizanidine, Levetiracetam, Fluoxetine,	Yes	33	Men	Control

			Amantadine, Alprazolam				
**26**	No-TBI	UWS	Trazodone, Baclofen, Levetiracetam, Clonazepam	No	66	Men	Control
**27**	TBI	UWS	Levetiracetam, Trazodone, Clonazepam, Baclofen, Gabapentin, Levodopa	Yes	20	Men	Control
**28**	No-TBI	UWS	Levetiracetam, Baclofen, Clonazepam	Yes	27	Men	Control
**29**	No-TBI	MCS	Baclofen, Levetiracetam	No	40	Men	Control
**30**	TBI	UWS	Baclofen, Clonazepam, Levetiracetam	No	12	Men	Control
**31**	TBI	UWS	Lacosamide, Perampanel, Levodopa, Amantadine	Yes	26	Men	Control
**32**	TBI	MCS	Baclofen, Lacosamide, Escitalopram	Yes	57	Women	Control
**33**	No-TBI	UWS	Baclofen	No	60	Women	Control
**34**	TBI	UWS	Baclofen, Levetiracetam	Yes	34	Women	Control
**35**	TBI	MCS	Baclofen, Levetiracetam	Yes	29	Women	Control
**36**	TBI	MCS	Baclofen, Levetiracetam, Clonazepam, Sertraline	Yes	53	Women	Control
**37**	TBI	UWS	Baclofen, Tizanidine	Yes	9	Men	Control
**38**	TBI	MCS	Citalopram, Clonazepam, Baclofen, Levetiracetam, Tizanidine, Lacosamide	No	26	Men	Control
**39**	TBI	MCS	Levetiracetam, Amantadine, Clonazepam, Escitalopram	Yes	72	Women	Control
**40**	No-TBI	UWS	Levetiracetam, Baclofen, Clonazepam, Trazodone	No	32	Women	Control
**41**	No-TBI	UWS	Trazodone, Baclofen, Amantadine	No	28	Men	Control
**42**	No-TBI	MCS	Levetiracetam, Clonazepam	No	25	Men	Control
**43**	TBI	MCS	Baclofen, Tizanidine, Levetiracetam, Lacosamide, Levodopa	Yes	25	Men	Control
**44**	TBI	MCS	Brivaracetam, Lacosamide, Baclofen, Quetiapine, Fluoxetine	Yes	18	Men	Control
**45**	-	-	-	-	27	Women	Healthy
**46**	-	-	-	-	48	Women	Healthy
**47**	-	-	-	-	32	Men	Healthy
**48**	-	-	-	-	21	Women	Healthy
**49**	-	-	-	-	23	Women	Healthy
**50**	-	-	-	-	56	Women	Healthy
**51**	-	-	-	-	32	Men	Healthy
**52**	-	-	-	-	27	Women	Healthy
**53**	-	-	-	-	27	Women	Healthy
**54**	-	-	-	-	25	Women	Healthy
**55**	-	-	-	-	42	Men	Healthy
**56**	-	-	-	-	38	Men	Healthy
**57**	-	-	-	-	23	Men	Healthy
**58**	-	-	-	-	25	Women	Healthy

**59**	-	-	-	-	41	Women	Healthy
**60**	-	-	-	-	46	Women	Healthy
**61**	-	-	-	-	25	Women	Healthy
**62**	-	-	-	-	34	Women	Healthy
**63**	-	-	-	-	22	Men	Healthy
**64**	-	-	-	-	45	Women	Healthy
**65**	-	-	-	-	24	Men	Healthy
**66**	-	-	-	-	33	Women	Healthy

Table S2. Timeframe of the study

Nº	Months between injury and first session	Days between first session and AVNT	Days between AVNT and second session	Days between sessions
**1**	8	0	18	43
**2**	15	4	22	56
**3**	6	21	8	59
**4**	9	7	2	38
**5**	9	27	10	62
**6**	7	4	21	55
**7**	21	0	3	33
**8**	5	7	0	36
**9**	12	12	0	42
**10**	7	19	0	48
**11**	12	0	13	43
**12**	5	14	6	48
**13**	15	0	7	44
**14**	11	4	3	42
**15**	9	18	0	48
**16**	9	2	15	120
**17**	5	6	14	49
**18**	7	62	22	114
**19**	9	13	7	49
**20**	12	0	4	34
**21**	3	12	7	56
**22**	9	6	17	45
**23**	6	-	-	132

**24**	4	-	-	59
**25**	6	-	-	31
**26**	7	-	-	53
**27**	3	-	-	63
**28**	4	-	-	14
**29**	7	-	-	60
**30**	3	-	-	85
**31**	5	-	-	21
**32**	2	-	-	51
**33**	5	-	-	20
**34**	6	-	-	38
**35**	5	-	-	61
**36**	13	-	-	48
**37**	8	-	-	62
**38**	12	-	-	45
**39**	3	-	-	200
**40**	4	-	-	26
**41**	15	-	-	146
**42**	3	-	-	182
**43**	18	-	-	61
**44**	4	-	-	48
**45**	-	-	-	145
**46**	-	-	-	125
**47**	-	-	-	203
**48**	-	-	-	249
**49**	-	-	-	203
**50**	-	-	-	35
**51**	-	-	-	57
**52**	-	-	-	121
**53**	-	-	-	63
**54**	-	-	-	243
**55**	-	-	-	235
**56**	-	-	-	115
**57**	-	-	-	41
**58**	-	-	-	49

**59**	-	-	-	181
**60**	-	-	-	147
**61**	-	-	-	15
**62**	-	-	-	208
**63**	-	-	-	44
**64**	-	-	-	146
**65**	-	-	-	41
**66**	-	-	-	8

Table S3. Mean retained epochs after artifact rejection for each group and condition.

		**Frequent**		**Infrequent**	
		**First session**	**Second session**	**First session**	**Second session**
**Experimental**	**Total**	128	131	43	43
	**MCS**	126	134	43	45
	**UWS**	134	125	44	43
	**TBI**	127	128	43	43
	**No-TBI**	130	135	44	44
**Control**	**Total**	127	128	45	42
	**MCS**	127	123	42	42
	**UWS**	127	131	47	43
	**TBI**	132	135	44	45
	**No-TBI**	120	118	48	38
**Healthy**		145	145	48	48

Table S4. Participants identified as outliers based on the classification and type of analysis performed, along with the sample size for each comparative group.

	Variable	Normalization	Outlier codes	Final sample size
Groups	Peak amplitude	No	60 and 66	Experimental → 22<br>Control → 22<br>Healthy → 20
		Yes	60	Experimental → 22<br>Control → 22<br>Healthy → 21
	Peak-to-Peak amplitude	No	58	Experimental → 22<br>Control → 22<br>Healthy → 21
		Yes	58	Experimental → 22<br>Control → 22<br>Healthy → 21
Diagnoses	Peak amplitude	No	24	Experimental with MCS → 15<br>Experimental with UWS → 7<br>Control with MCS → 9<br>Control with UWS → 12
		Yes	-	Experimental with MCS →15<br>Experimental with UWS → 7<br>Control with MCS → 9<br>Control with UWS → 13
	Peak-to-Peak amplitude	No	-	Experimental with MCS → 15<br>Experimental with UWS → 7<br>Control with MCS → 9<br>Control with UWS → 13
		Yes	-	Experimental with MCS → 15<br>Experimental with UWS → 7<br>Control with MCS → 9<br>Control with UWS → 13
Etiologies	Peak amplitude	No	3	Experimental with TBI → 12<br>Experimental with No-TBI → 9<br>Control with TBI → 13<br>Control with No-TBI → 9
		Yes	3	Experimental with TBI → 12<br>Experimental with No-TBI → 9<br>Control with TBI → 13<br>Control with No-TBI → 9
	Peak-to-Peak amplitude	No	-	Experimental with TBI → 13<br>Experimental with No-TBI → 9<br>Control with TBI → 13<br>Control with No-TBI → 9
		Yes	-	Experimental with TBI → 13<br>Experimental with No-TBI → 9<br>Control with TBI → 13<br>Control with No-TBI → 9

Note: The outlier codes refer to the anonymized identification numbers of the participants.

Section S3 (Supplementary results)

This section reports the results of unnormalized analyses of MMN peak and peak-to-peak amplitude, stratified by groups, diagnosis and etiologies.

Section S3.1. Classification by groups

Section S3.1.1. Peak amplitude

No significant differences between sessions were found for any comparative group, according to the Wilcoxon test. Similarly, no significant differences were observed between groups during the first session, as assessed by the Kruskal-Wallis test. However, the Friedman test revealed topographical differences only in the healthy group [$Q(3) = 19.44$, $p < 0.001$], with the fronto-central [$W = 17$, $p_{Bonferroni} = 0.002$, $d = 0.910$, $r = 0.734$], left [$W = 9$, $p_{Bonferroni} < 0.001$, $d = 1.101$, $r = 0.801$], and right [$W = 7$, $p_{Bonferroni} < 0.001$, $d = 1.016$, $r = 0.818$] regions exhibiting more negative amplitudes than the parieto-occipital region.

Conversely, the Kruskal-Wallis test reached significance only during the second session for the parieto-occipital [$H(2) = 6.81$, $p = 0.033$], left [$H(2) = 9.74$, $p = 0.008$], and right [$H(2) = 8.41$, $p = 0.015$] regions. In this case, the healthy group showed higher negative amplitudes than the control group in the parieto-occipital [$U = 319$, $p_{Bonferroni} = 0.039$, $d = 0.848$, $r = 0.388$], left [$U = 342$, $p_{Bonferroni} = 0.007$, $d = 1.064$, $r = 0.474$], and right [$U = 342$, $p_{Bonferroni} = 0.007$, $d = 0.968$, $r = 0.474$] regions, while no significant differences were observed with the experimental group. Additionally, the topographical differences detected in the first session were no longer present.

Unnormalized amplitude values for each group are presented in Table S5.

Table S5. Mean peak amplitude of the MMN component.

	Session	Fronto-Central ($\bar{x}$ ± SD µV)	Parieto-Occipital ($\bar{x}$ ± SD µV)	Left ($\bar{x}$ ± SD µV)	Right ($\bar{x}$ ± SD µV)
**Experimental**	First session	-1.39 ± 3.84	-0.66 ± 3.99	-0.83 ± 4.47	-1.26 ± 4.48
	Second session	-1.86 ± 4.64	-1.35 ± 3.37	-1.32 ± 3.61	-2.39 ± 5.18
**Control**	First session	-0.74 ± 2.77	-0.64 ± 4.83	-0.90 ± 4.38	-1.04 ± 2.70
	Second session	-0.27 ± 4.46	0.27 ± 3.90 (#)	0.25 ± 3.66 (#)	-0.03 ± 3.55 (#)
**Healthy**	First session	-2.58 ± 3.65	-1.53 ± 3.46 (†)	-2.43 ± 3.53	-2.65 ± 3.20
	Second session	-3.33 ± 3.64	-2.78 ± 3.23 (#)	-3.50 ± 3.37 (#)	-3.34 ± 3.25 (#)

Note: The asterisk symbol (*) represents significant differences between sessions (Wilcoxon test), whereas the hashtag symbol (#) indicates significant differences between groups (Kruskal–Wallis and Mann–Whitney U tests) and the dagger symbol (†) marks differences between regions (Friedman and Wilcoxon tests). The significance level for all tests was set at $p_{Bonferroni} < 0.05$.

Section S3.1.2. Peak-to-Peak amplitude

The Wilcoxon test revealed that only the control group exhibited a significant decrease in the peak-to-peak amplitude of the MMN component during the second session in the right region [$W(21) = 56$, $p = 0.021$, $d = 0.546$, $r = 0.488$]. Furthermore, Kruskal-Wallis

[$H(2) = 6.48$, $p = 0.039$] and Mann-Whitney U [$U = 131$, $p_{Bonferroni} = 0.047$, $d = 0.811$, $r = 0.371$] tests indicated that this decrease led to the control group showing significantly lower amplitudes than the healthy group during the second session.

According to the Friedman test, only the healthy group during the first session showed significant topographical differences [$Q(3) = 16.26$, $p = 0.001$], with the parieto-occipital region exhibiting greater amplitude than the fronto-central [$W = 30$, $p_{Bonferroni} = 0.011$, $d = 0.758$, $r = 0.648$], left [$W = 21$, $p_{Bonferroni} = 0.003$, $d = 0.921$, $r = 0.717$] and right [$W = 16$, $p_{Bonferroni} < 0.001$, $d = 0.835$, $r = 0.755$] regions.

Peak-to-peak amplitude values of the MMN component by groups are presented in Table S6.

Table S6. Mean peak-to-peak amplitude of the MMN component

	Session	Fronto-Central ($\bar{x} \pm$ SD μV)	Parieto-Occipital ($\bar{x} \pm$ SD μV)	Left ($\bar{x} \pm$ SD μV)	Right ($\bar{x} \pm$ SD μV)
**Experimental**	First session	2.46 ± 1.94	2.29 ± 2.24	2.69 ± 2.17	2.40 ± 2.04
	Second session	3.11 ± 2.00	2.77 ± 2.18	3.44 ± 3.12	3.31 ± 3.08
**Control**	First session	2.88 ± 2.34	2.92 ± 2.30	2.91 ± 2.21	2.95 ± 1.78 (*)
	Second session	2.88 ± 2.40	1.85 ± 1.45	2.21 ± 1.56	1.79 ± 1.18 (*)(#)
**Healthy**	First session	3.65 ± 2.22	2.15 ± 1.27 (†)	3.04 ± 1.44	3.00 ± 1.67
	Second session	3.54 ± 2.21	2.27 ± 1.30	2.97 ± 1.56	2.89 ± 1.51 (#)

Note: The asterisk symbol (*) represents significant differences between sessions (Wilcoxon test), whereas the hashtag symbol (#) indicates significant differences between groups (Kruskal–Wallis and Mann–Whitney U tests) and the dagger symbol (†) marks differences between regions (Friedman and Wilcoxon tests). The significance level for all tests was set at $p_{Bonferroni} < 0.05$.

Section S3.2. Classification by diagnoses

Section S3.2.1. Peak amplitude

The Wilcoxon test revealed that participants diagnosed with UWS in the control group exhibited during the second session a shift of the MMN from negative to positive amplitudes across fronto-central [$W(11) = 11$, $p = 0.027$, $d = 0.750$, $r = 0.634$], parieto-occipital [$W(11) = 1$, $p = 0.001$, $d = 0.774$, $r = 0.861$], left [$W(11) = 9$, $p = 0.016$, $d = 0.845$, $r = 0.679$], and right [$W(11) = 2$, $p = 0.001$, $d = 1.184$, $r = 0.838$] regions (Table S7). No other significant differences were observed either between or within groups, nor were there any significant topographical differences.

Table S7. Mean peak amplitude of the MMN component, by diagnosis.

	Session	Fronto-Central ($\bar{x}$ ± SD μV)	Parieto-Occipital ($\bar{x}$ ± SD μV)	Left ($\bar{x}$ ± SD μV)	Right ($\bar{x}$ ± SD μV)
**Experimental with UWS**	First session	-0.66 ± 3.55	-0.07 ± 3.86	-0.61 ± 4.23	-0.20 ± 3.37
	Second session	-1.92 ± 5.50	-0.48 ± 4.63	0.50 ± 4.11	-1.56 ± 6.49
**Experimental with MCS**	First session	-1.73 ± 4.04	-0.93 ± 4.15	-0.93 ± 4.72	-1.75 ± 4.93
	Second session	-1.83 ± 4.40	-1.75 ± 2.69	-2.17 ± 3.14	-2.77 ± 4.66
**Control with UWS**	First session	-1.25 ± 2.08 (*)	-2.12 ± 3.24 (*)	-1.96 ± 2.47 (*)	-1.67 ± 2.11 (*)
	Second session	1.86 ± 3.78 (*)	1.36 ± 2.69 (*)	1.50 ± 2.23 (*)	1.61 ± 1.51 (*)
**Control with MCS**	First session	-0.25 ± 3.62	-0.16 ± 4.60	-0.93 ± 4.40	-0.61 ± 3.24
	Second session	-2.43 ± 4.00	-0.13 ± 4.13	-0.55 ± 4.21	-1.44 ± 4.19

Note: The asterisk symbol (*) represents significant differences between sessions (Wilcoxon test), whereas the hashtag symbol (#) indicates significant differences between groups (Kruskal–Wallis and Mann–Whitney U tests) and the dagger symbol (†) marks differences between regions (Friedman and Wilcoxon tests). The significance level for all tests was set at $p_{Bonferroni} < 0.05$.

Section S3.2.2. Peak-to-Peak amplitude

No significant differences were detected either between diagnoses or between sessions in the peak-to-peak analysis, nor were there any notable topographical differences (Table S8).

Table S8. Mean peak-to-peak amplitude of the MMN component, by diagnosis.

	Session	Fronto-Central ($\bar{x}$ ± SD μV)	Parieto-Occipital ($\bar{x}$ ± SD μV)	Left ($\bar{x}$ ± SD μV)	Right ($\bar{x}$ ± SD μV)
**Experimental with UWS**	First session	2.19 ± 1.92	2.13 ± 2.53	2.61 ± 2.82	1.86 ± 1.65
	Second session	1.86 ± 1.10	2.20 ± 1.77	1.79 ± 1.21	2.53 ± 1.83
**Experimental with MCS**	First session	2.59 ± 2.01	2.36 ± 2.19	2.73 ± 1.91	2.65 ± 2.20
	Second session	3.70 ± 2.07	3.04 ± 2.35	4.21 ± 3.46	3.67 ± 3.51
**Control with UWS**	First session	2.83 ± 2.22	3.24 ± 2.83	2.90 ± 1.40	2.86 ± 1.83
	Second session	2.77 ± 2.20	1.97 ± 1.37	2.20 ± 1.15	1.94 ± 1.07
**Control with MCS**	First session	2.94 ± 2.65	2.47 ± 1.20	2.93 ± 3.14	3.09 ± 1.81
	Second session	3.03 ± 2.80	1.67 ± 1.62	2.24 ± 2.10	1.58 ± 1.36

Note: The asterisk symbol (*) represents significant differences between sessions (Wilcoxon test), whereas the hashtag symbol (#) indicates significant differences between groups (Kruskal–Wallis and Mann–Whitney U tests) and the dagger symbol (†) marks differences between regions (Friedman and Wilcoxon tests). The significance level for all tests was set at $p_{Bonferroni} < 0.05$.

Section S3.3. Classification by etiologies

Section S3.3.1. Peak amplitude

No significant differences were found within or between etiologies, according to the Wilcoxon and Kruskal–Wallis tests. However, only topographical differences were detected in the Friedman test for the participants with TBI in the experimental group during the first session [$Q(3) = 10.90, p = 0.012$], in which the fronto-central region showed a higher negative amplitude than the right region [$W = 6, p_{Bonferroni} = 0.041, d = 0.813, r = 0.747$] (Table S9).

Table S9. Mean peak amplitude of the MMN component, by etiologies.

	Session	Fronto-Central ($\bar{x}$ ± SD μV)	Parieto-Occipital ($\bar{x}$ ± SD μV)	Left ($\bar{x}$ ± SD μV)	Right ($\bar{x}$ ± SD μV)
**Experimental with No-TBI**	First session	0.15 ± 3.68	0.22 ± 4.02	0.24 ± 4.73	-0.25 ± 3.23
	Second session	-1.17 ± 4.03	-0.50 ± 3.30	0.33 ± 3.54	-1.30 ± 4.55
**Experimental with TBI**	First session	-1.79 ± 2.94 (†)	-0.48 ± 2.96	-0.71 ± 3.17	-0.68 ± 2.52 (†)
	Second session	-2.36 ± 5.35	-1.62 ± 3.38	-2.27 ± 3.38	-3.00 ± 5.86
**Control with No-TBI**	First session	0.08 ± 2.06	-0.79 ± 6.10	0.39 ± 4.83	-1.25 ± 2.66
	Second session	-0.50 ± 3.00	1.19 ± 4.87	0.47 ± 4.13	0.40 ± 3.44
**Control with TBI**	First session	-1.30 ± 3.13	-0.55 ± 4.00	-1.80 ± 3.99	-0.89 ± 2.82
	Second session	-0.11 ± 5.36	-0.36 ± 3.11	0.10 ± 3.47	-0.33 ± 3.74

Note: The asterisk symbol (*) represents significant differences between sessions (Wilcoxon test), whereas the hashtag symbol (#) indicates significant differences between groups (Kruskal–Wallis and Mann–Whitney U tests) and the dagger symbol (†) marks differences between regions (Friedman and Wilcoxon tests). The significance level for all tests was set at $p_{Bonferroni} < 0.05$.

Section S3.3.2. Peak-to-Peak amplitude

Participants with TBI in the control group showed a decrease in peak-to-peak MMN amplitude in the right region during the second session [$W(12) = 16, p = 0.040, d = 0.726, r = 0.572$], as indicated by the Wilcoxon test. Furthermore, Kruskal-Wallis [$H(3) = 10.52, p = 0.015$] and Mann-Whitney U tests revealed that TBI participants in the control group exhibited lower peak-to-peak amplitudes than those in the experimental group in the right region during the second session [$U = 143, p_{Bonferroni} = 0.018, d = 1.034, r = 0.588$]. No differences in topographic distribution were observed (Table S10).

Table S10. Mean peak-to-peak amplitude of the MMN component, by etiologies.

	Session	Fronto-Central ($\bar{x}$ ± SD μV)	Parieto-Occipital ($\bar{x}$ ± SD μV)	Left ($\bar{x}$ ± SD μV)	Right ($\bar{x}$ ± SD μV)
**Experimental with No-TBI**	First session	1.95 ± 1.82	1.93 ± 2.12	2.42 ± 2.43	2.00 ± 1.30
	Second session	2.07 ± 1.87	1.99 ± 2.22	2.04 ± 2.03	2.04 ± 2.25
**Experimental with TBI**	First session	2.82 ± 2.02	2.53 ± 2.38	2.88 ± 2.04	2.68 ± 2.44
	Second session	3.84 ± 1.81	3.31 ± 2.06	4.41 ± 3.44	4.19 ± 3.34 (#)
**Control with No-TBI**	First session	2.57 ± 2.19	3.54 ± 3.35	2.81 ± 1.41	2.77 ± 1.91
	Second session	3.06 ± 2.37	1.66 ± 1.13	2.24 ± 0.98	2.08 ± 1.18
**Control with TBI**	First session	3.10 ± 2.51	2.50 ± 1.13	2.98 ± 2.68	3.08 ± 1.76 (*)
	Second session	2.75 ± 2.51	1.98 ± 1.67	2.19 ± 1.90	1.60 ± 1.18 (*)(#)

Note: The asterisk symbol (*) represents significant differences between sessions (Wilcoxon test), whereas the hashtag symbol (#) indicates significant differences between groups (Kruskal–Wallis and Mann–Whitney U tests) and the dagger symbol (†) marks differences between regions (Friedman and Wilcoxon tests). The significance level for all tests was set at $p_{Bonferroni} < 0.05$.

Section S4. Additional demographic analyses

The healthy group had a significantly higher proportion of women compared to the control and experimental groups [$\chi^2(2, 66) = 9.80$, $p = 0.007$]. Although no significant sex differences were observed for any MMN variables after correction for multiple comparisons in unnormalised measures or in normalised peak-to-peak amplitude and latency measures, significant sex-related differences were identified in normalised peak amplitude measures. In this case, males and females differed significantly in the first session [fronto-central (U = 336, p = 0.011, d = 0.792, r = 0.368); parieto-occipital (U = 299, p = 0.002, d = 0.974, r = 0.438); left (U = 330, p = 0.008, d = 0.822, r = 0.380); right (U = 357, p = 0.023, d = 0.697, r = 0.329)], the second session [fronto-central (U = 828, p < 0.001, d = 1.338, r = 0.556); parieto-occipital (U = 784, p < 0.001, d = 1.077, r = 0.474); left (U = 836, p < 0.001, d = 1.391, r = 0.571); right (U = 857, p < 0.001, d = 1.544, r = 0.611)], and the analysis of inter-session changes [fronto-central (U = 840, p < 0.001, d = 1.420, r = 0.578); parieto-occipital (U = 868, p < 0.001, d = 1.631, r = 0.632); left (U = 837, p < 0.001, d = 1.398, r = 0.573); right (U = 839, p < 0.001, d = 1.413, r = 0.577)]. No significant differences in diagnosis or etiology were observed between clinical groups.
